# Recent expansion and adaptive evolution of the carcinoembryonic antigen family in bats of the Yangochiroptera subgroup

**DOI:** 10.1186/s12864-017-4106-7

**Published:** 2017-09-11

**Authors:** Robert Kammerer, Martin Mansfeld, Jana Hänske, Sophie Mißbach, Xiaocui He, Bernd Köllner, Susan Mouchantat, Wolfgang Zimmermann

**Affiliations:** 1 Institute of Immunology, Friedrich-Loeffler Institute, -Insel Riems, Greifswald, Germany; 2 Plattform Degenerative Erkrankungen, Deutsches Primatenzentrum GmbH, Goettingen, Germany; 3 Department of Molecular Immunology, Max-Planck-Institute of Immunobiology and Epigenetics, Freiburg, Germany; 4 Junior Research Group Wildlife Diseases, Friedrich-Loeffler-Institute, -Insel Riems, Greifswald, Germany; 5 Institute of Diagnostic Radiology and Neuroradiology, University Medicine Greifswald, Greifswald, Germany; 6 Tumor Immunology Laboratory, LIFE Center, University Clinic, Ludwig-Maximilians-University, Munich, Germany; 7 Department of Urology, University Clinic, Ludwig-Maximilians-University, Munich, Germany

**Keywords:** Carcinoembryonic antigen gene family, Pregnancy-specific glycoproteins, Positive selection, Chiroptera, Immunoglobulin superfamily, Bats

## Abstract

**Background:**

Expansions of gene families are predictive for ongoing genetic adaptation to environmental cues. We describe such an expansion of the *carcinoembryonic antigen* (*CEA*) gene family in certain bat families. Members of the CEA family in humans and mice are exploited as cellular receptors by a number of pathogens, possibly due to their function in immunity and reproduction. The CEA family is composed of CEA-related cell adhesion molecules (CEACAMs) and secreted pregnancy-specific glycoproteins (PSGs). PSGs are almost exclusively expressed by trophoblast cells at the maternal-fetal interface. The reason why PSGs exist only in a minority of mammals is still unknown.

**Results:**

Analysis of the CEA gene family in bats revealed that in certain bat families, belonging to the subgroup Yangochiroptera but not the Yinpterochiroptera subgroup an expansion of the CEA gene family took place, resulting in approximately one hundred CEA family genes in some species of the Vespertilionidae. The majority of these genes encode secreted PSG-like proteins (further referred to as PSG). Remarkably, we found strong evidence that the ligand-binding domain (IgV-like domain) of PSG is under diversifying positive selection indicating that bat PSGs may interact with structurally highly variable ligands. Such ligands might represent bacterial or viral pathogen adhesins. We have identified two distinct clusters of PSGs in three Myotis species. The two PSG cluster differ in the amino acids under positive selection. One cluster was only expanded in members of the Vespertilionidae while the other was found to be expanded in addition in members of the Miniopteridae and Mormoopidae. Thus one round of PSG expansion may have occurred in an ancestry of all three families and a second only in Vespertilionidae. Although maternal ligands of PSGs may exist selective challenges by two distinct pathogens seem to be likely responsible for the expansion of PSGs in Vespertilionidae.

**Conclusions:**

The rapid expansion of PSGs in certain bat species together with selection for diversification suggest that bat PSGs could be part of a pathogen defense system by serving as decoy receptors and/or regulators of feto-maternal interactions.

**Electronic supplementary material:**

The online version of this article (10.1186/s12864-017-4106-7) contains supplementary material, which is available to authorized users.

## Background

Gene families are predestinated to rapid genetic adaptation to environmental cues in vertebrates. Accelerated gene family expansion, therefore, may provide hints for environmental forces on vertebrate species. For example, in species with an extraordinary large number of γ/δ T-cells, like cattle, sheep and chicken, the CD163 family of immune receptors which are pivotal for the function of γ/δ T-cells is expanded [1]. These species are not very closely related, indicating that the evolution of this gene family is not due to an expansion in a common ancestor but happened independently probably driven by pathogens [1]. Such a species-specific expansion is seen in various gene families, most of them being involved in immunity and/or reproduction. However, the precise function of members of these gene families is often unknown.

This is also true for the *carcinoembryonic antigen* (*CEA*) gene family, which belongs to the immunoglobulin superfamily and represents one of the fastest evolving gene families in mammals [2]. In mammals, the ancestral *CEA* gene family was composed of five genes, i.e. *CEACAM1, CEACAM16, CEACAM18, CEACAM19* and *CEACAM20*. These genes can be identified in almost all mammalian species. The ancestral *CEACAM1* was subject to multiple duplications which led to species-specific expansion of *CEACAM1*-related members of the *CEA* gene family. CEACAM1 is a transmembrane inhibitory receptor composed of one N-terminal immunoglobulin variable (IgV)-like (also called N domain) and three Ig constant (IgC)-like extracellular domains (also named A1, B, and A2 domains). The IgV-like domain is the primary ligand-binding domain, which was shown to interact with other CEACAMs and other cell surface receptors such as galectins, integrins and TIM-3 as well as with various pathogen adhesins [3, 4]. The cytoplasmic tail of CEACAM1 contains one to two immunoreceptor tyrosine-based inhibition motifs (ITIM). CEACAM1 is expressed by various cell types including, endothelial, epithelial and immune cells. In immune cells CEACAM1 is an important regulator of cell activation [5–7]. In primates and rodents the *CEACAM1*-related genes belong either to the *CEA-related cell adhesion molecule* (*CEACAM*) or the *pregnancy-specific glycoprotein* (*PSG*) subgroups. While several CEACAMs are receptors involved in immunity, PSGs are expressed nearly exclusively in trophoblast cells and most likely play a role in maternal-fetal communication [8]. Surprisingly, PSGs do not exist in various mammals including most of the members of the superorder Laurasiatheria [9–11]. However, more recently we and others found that in bats, namely in *Myotis lucifugus* (*M. lucifugus*) and *Myotis davidii* (*M. davidii*) which also belong to the superorder Laurasiatheria, a considerable gene amplification in the *CEA* gene family occurred [11, 12]. However, information on the structure and expression of the CEA family members in bats are completely missing.

Bats belong to the order Chiroptera, which is the second largest order of mammals, only rodents contain more species. Traditionally the order Chiroptera was divided into the two suborders Megachiroptera (fruit-eating, non-echolocating bats) and Microchiroptera (insectivorous, echolocating bats). However, current molecular evidence rather favors the division in the new subgroups Yinpterochiroptera and Yangochiroptera which have diverged approximately 60 million years ago [13, 14]. Yinpterochiroptera contain in addition to the old world fruit bats (Pteropodidae), four families of echolocating insectivorous bats. Common to all bats is that they play an important role as reservoirs for viruses. Currently more than 100 viruses have been detected in bats some of them, like lyssa, corona and ebola viruses, are of extraordinary importance for human health [15]. As a consequence it has been speculated that the immune system of bats has unique features making them tolerant to several virus infections [16]. Indeed the continuous threat by various pathogens may have a strong influence on the evolution of immune proteins, including the CEACAM receptors of the CEA family. In addition, we have recently speculated that the expansion of the *PSG* subgroup of the *CEA* gene family requires the presence of a hemochorial placenta as it is found in primates and rodents. In contrast to endotheliochorial and epitheliochorial placentae the hemochorial placenta allows direct contact of fetal cells with maternal blood and immune cells [17]. Bats have either an endotheliochorial or a hemochorial placenta, depending on the bat species. Therefore, the high number of CEACAMs in certain bat species raises the question whether these CEACAMs may represent PSGs.

In this report we show that the vast majority of CEACAMs in bats, which have an extended CEA family, are secreted glycoproteins. These glycoproteins are not expressed in a number of tissues in which usually non-PSG CEACAMs are expressed suggesting that the secreted CEACAMs in bats have a restricted expression pattern. Interestingly, the IgV-like domain which is responsible for the interaction with almost all extracellular ligands is under strong positive selection in bats. Selection for diversification points to rapidly evolving ligands, like viruses and other microorganisms or to a family of closely related receptors, like members of a protein family. We hypothesize that these PSG-like proteins (further referred to as PSGs) are expressed at the maternal-fetal interface and that they play a role either in counteracting infection or regulating maternal-fetal communication.

## Results

### Phylogeny of bat orthologous *CEACAM* genes

**Fig. 1:**
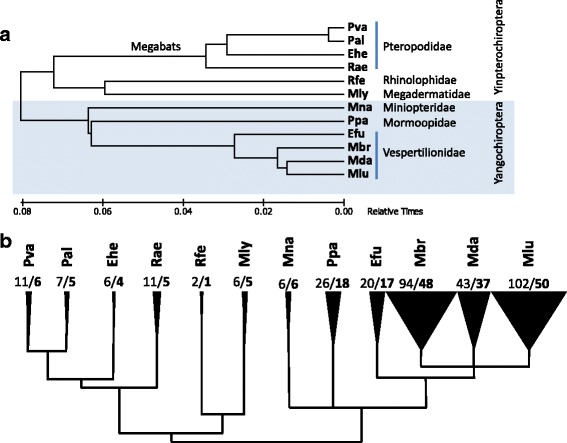
The CEA gene family in microbats is the most populous so far found in mammals. **a** The phylogenetic tree of analyzed bats consisting of 12 species and six families was constructed using the nucleotide sequence of the exons coding for the extracellular domains of CEACAM16, CEACAM18 and CEACAM19. Two main bat subgroups belonging to either Yinpterochiroptera or Yangochiroptera suborders (marked in blue) were identified. Each suborder contains six species belonging to three bat families each. **b** The expansion of CEACAM1-like genes in different bat species is depicted. Numbers indicate the number of N domain exons found in the indicated species: total number of N domain exons/number of N domain exons with ORF (bold). The base of the triangles is proportional to the total number of CEA gene family members. Cut-off level for condensed tree is 50%. Efu, *Eptesicus fuscus*; Ehe, *Eidolon helvum*; Mbr, *Myotis brandtii*; Mda, *Myotis davidii*; Mlu, *Myotis lucifugus;* Mly, *Megaderma lyra*; Mna, *Miniopterus natalensis*; Pal, *Pteropus alecto*; Ppa, *Pteronotus parnellii*; Pva, *Pteropus vampyrus*; Rae, *Rousettus aegyptiacus*; Rfe, *Rhinolophus ferrumequinum*

First we searched for bat orthologous *CEACAM* genes *CEACAM16*, *CEACAM18* and *CEACAM19* in the “whole-genome shotgun contigs (wgs)” database at NCBI using cDNA sequences of individual exons of human orthologous genes. Significant hits (E value < e-20; Query cover >50%) were obtained for 12 bat species belonging to six families. Six species belong to the Yangochiroptera and six species to the Yinpterochiroptera subgroup including four megabat species. Using sequences coding for extracellular domains (CEACAM16N1, A, B, N2, CEACAM18N, CEACAM19N) of orthologous CEACAMs we could identify all orthologous *CEACAM* genes in each species, except for *M. lucifugus* for which we did not find *CEACAM16*. The retrieved sequences were concatenated to construct a phylogenetic tree of these bat species (Fig. 1a). The phylogenetic tree based on orthologous *CEACAM* sequences, depicted very closely the phylogenetic relationship of bat families published previously based on other genetic data except for the relative position of Mormoopidae and Miniopteridae to Vespertilionidae [14, 18]. While Miniopteridae and Vespertilionidae are considered to belong to the Vespertilionoides superfamily, Mormoopidae belong to the Noctilionoidea superfamily. However, similar difficulties were reported by Agnarsson and colleagues who build a phylogenetic tree based on the single cytochrome b gene [19].

### Tremendous expansion of the *CEA* gene family in certain bat species of the Yangochiroptera subgroup

Next we determined the number of CEACAM1 paralogs in each bat species as described in “Materials and Methods”. Sequences without an open reading frame (ORF) were considered to be part of a pseudogene. Within Yinpterochiroptera maximally 11 N domain exon sequences were identified per species with a maximum of six N domain exons with an ORF within one species (Fig. 1b). *Miniopterus natalensis* (*M. natalensis*) a species of the Yangochiroptera suborder has also six N domain exon sequences with an ORF. In all other species of the Yangochiroptera group investigated, a tremendous expansion of *CEA* family member N domain exons was observed. In *M. lucifugus* 102 different N domain exons were found, nearly half of them contained an ORF (Fig. 1b). Roughly, a one to one ratio of N domain exons with internal stop codons and N domain exons with an ORF were also found in *Myotis brandii* (*M. brandii*), while in *M. davidii*, *Eptesicus fuscus* (*E. fuscus*) and *Pteronotus parnellii* (*P. parnellii*) N domain exon sequences with internal stop codons were less frequent (Fig. 1b). Importantly, every N domain exon sequence was separated from another N domain sequence by *CEA* family-related exon sequences encoding other domains, strongly indicating that in bats each *CEA* family gene contains only a single N domain exon. Thus the number of N domain exons may indicate the number of *CEA* gene family members in bats.

### A balanced expansion of genes coding for inhibitory and activation CEACAM receptors frequently took place in bats

**Fig. 2:**
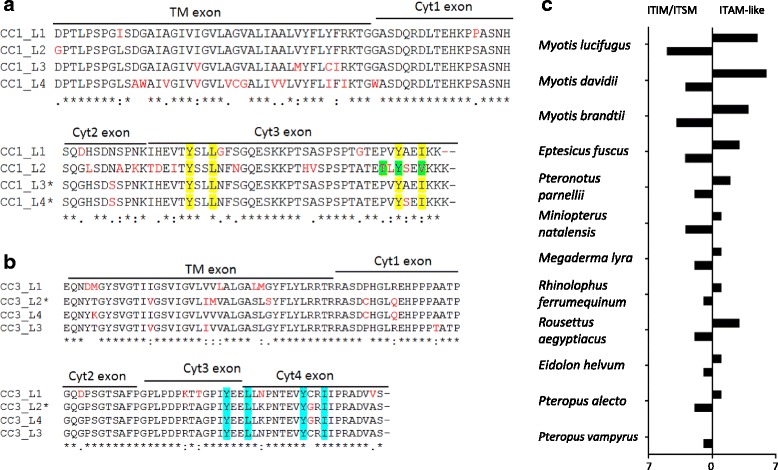
A balanced expansion of inhibitory and activating receptors occurred in bat species. Amino acid sequences of transmembrane and cytoplasmic domains of the CEA-related CEACAMs in *M. lucifugus* were aligned using ClustalW. **a** Sequences derived from inhibitory receptors. Amino acids which differ from the CEACAM1-like consensus sequence were marked in red. The immunoreceptor tyrosine-based inhibition motif (ITIM) and immunoreceptor tyrosine-based switch motif (ITSM) are highlighted in yellow and green, respectively. **b** Amino acid sequences from CEACAMs having an immunoreceptor tyrosine-based activation motif (ITAM) motif in the cytoplasmic tail. Amino acids which differ from the CEACAM3-like consensus sequence were marked in red. ITAM motifs are highlighted in blue. **a** and **b** Sequences marked with asterisks are derived from genes which have mutated splice donor sites between cytoplasmic exons. **c** The number of transmembrane domains associated with ITIM and ITAM signaling motifs of all bat species analyzed are depicted. Cyt, cytoplasmic; TM, transmembrane

Members of the CEA family may be secreted or membrane-bound glycoproteins. Membrane anchorage is accomplished by the presence of one of two types of transmembrane domain exons. One is derived from an ancestral *CEACAM1* gene and is combined with exons encoding a cytoplasmic tail containing inhibitory signaling motifs, and the second is derived from an ancestral *CEACAM* gene which had exons encoding an activation signaling motif [9]. The function of CEACAMs largely depends on their type of membrane anchorage and their signaling capacity. Using nucleotide sequences from human *CEACAM1* (encodes an inhibitory receptor) and human *CEACAM3* (encodes an activating/endocytic receptor) transmembrane domain exons we searched for related sequences within the *M. lucifugus* genome. We were able to identify five sequences which were related to the transmembrane domain exon of *CEACAM1* and five sequences which were related to the transmembrane domain exon of *CEACAM3*. Four sequences were next to exons encoding cytoplasmic tails with either two ITIM (three genes) or one ITIM and one immunoreceptor tyrosine-based switch motif (ITSM; one gene) while four transmembrane domain exons were coupled with cytoplasmic domain exons coding for immunoreceptor tyrosine-based activation motifs (ITAM). However, in three of these genes (two encoding ITIM and one ITAM) one splice donor site each is mutated indicating that these genes do not code for functional immunoreceptor tyrosine-based signaling motifs (Fig. 2a, b). For two of these genes the corresponding N domain sequence could not be identified. As shown in Fig. 2c such a balanced expansion took place in 50% of the bat species analyzed. The relatively small fraction of sequences that code for transmembrane domains (10 out of 102) in *M. lucifugus* was surprising and indicates that the majority of CEA family members in that species are not fixed to the cell membrane.

### Genes encoding secreted CEA family members expanded in bat species of the Yangochiroptera suborder

**Fig. 3:**
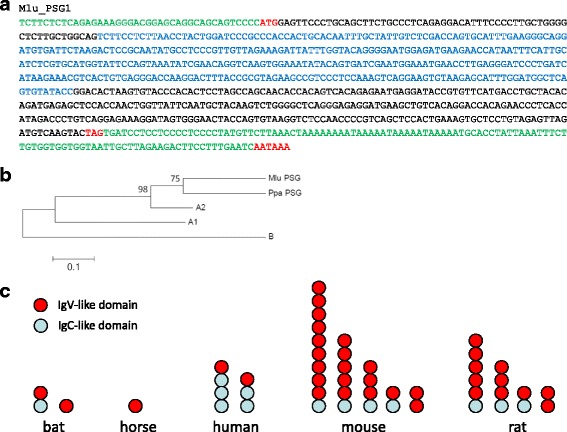
Structure of bat PSGs. **a** The exon structure of a typical bat PSG mRNA, composed of a leader sequence (black), an IgV-like domain exon (blue) and an IgC-like domain exon (black), is shown. 5′-UTR and 3′-UTR are shown in green. The start codon, the stop codon and the polyadenylation signals are shown in red. The stop codon in Mlu_*PSG1* is located at the end of the A domain exon. In other bat *PSGs* the stop codon is also in the A domain exons at varying positions (not shown). **b** Nucleotide sequences of the IgC-like domains of one representative PSG each from *M. lucifugus* and *P. parnellii* were aligned with the sequences of IgC-like domains of CEACAM1 from *M. lucifugus* (A1, B and A2)*.* The relationship of the sequences is depicted as a rooted dendrogram which was calculated using the MEGA5 software. The statistical support for each node is expressed as bootstrap values. The bar below the phylogenetic tree shows the scale for the number of substitutions per site. **c** Comparison of the domain organization of microbat PSGs with PSGs in primates, rodents and horse. IgV-like domains are depicted in red and IgC-like domains in blue

The predicted exon composition and order of most bat *CEA* gene family members without a transmembrane domain exon is as follows: a leader, IgV-like domain, and one IgC-like domain exon. In all of these genes, the latter exon has an internal stop codon either at the end of the IgC-like domain exon (PSGs with two immunoglobulin domains) or somewhere within the IgC-like domain (PSGs with only one immunoglobulin domain (Fig. 3a). Comparison of the IgC-like domains of secreted CEACAMs from *M. lucifugus* and *P. parnellii* with the IgC domains of CEACAM1 of *M. lucifugus* (i.e. A1, B, A2) revealed that they are most closely related with the A2-type IgC-like domains (Fig. 3b). Such exon arrangement and stop codon localization as found in the one domain PSGs was previously also found in equine PSGs (Fig. 3c). N glycosylation sites of the IgV-like domains varied between zero and two. This indicates that the majority of CEA gene family members in certain Yangochiroptera species codes for secreted proteins.

### PSG N domains are more closely related with each other than with N domains of membrane anchored CEACAMs in *M. lucifugus*

**Fig. 4:**
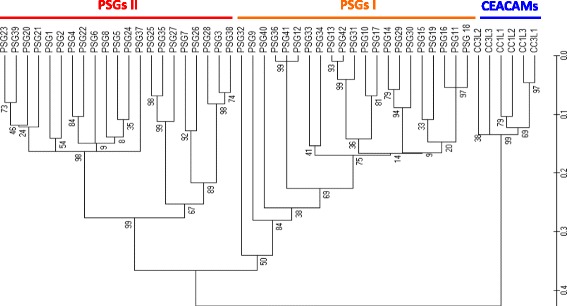
Relationship of CEACAM and PSG N domain sequences of *M. lucifugus.* Phylogenetic analysis of the IgV-like N domain amino acid sequences with open reading frames from the CEACAM1-like family members of *M. lucifugus*. CEACAMs without a transmembrane domain were named PSGs, according to the nomenclature of the human CEA family. Two separated groups of PSGs (PSG I and PSG II) with a similar number of members evolved in *M. lucifugus*. The relationship of the amino acid sequences is depicted as a rooted dendrogram using the MEGA5 software. The statistical support for each node is expressed as bootstra*p* values. The bar to the right of the phylogenetic tree shows the scale for the number of substitutions per site. CC1L1, CEACAM1-like_1; CC3L1, CEACAM3-like_1

Next we wanted to know how the presumed ligand-binding domains (the IgV-like domains) of the CEA family members in *M. lucifugus* are related. In humans, the ligand-binding domains of PSGs form a separate cluster within the CEA family. In *M. lucifugus* phylogenetic studies revealed that the ligand-binding domains form also two clusters separating the secreted PSGs and the transmembrane-anchored CEACAMs (Fig. 4). Interestingly, the ligand-binding domains of PSG in *M. lucifugus* formed two separated clusters further referred to as PSG I and PSG II.

### PSG N domains of Yangochiroptera bat species

**Fig. 5:**
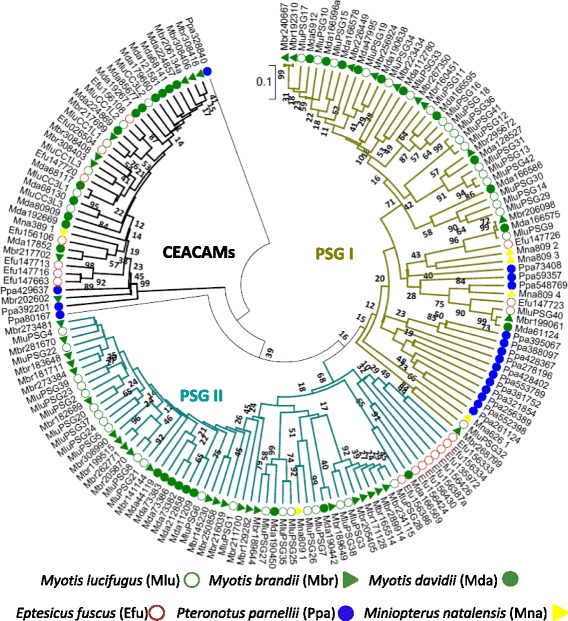
Variations in the *CEA* subfamily expansion in different microbat species indicate rapid evolution. Evolutionary relationship of amino acid sequences of the N domains of microbats. The phylogenetic tree illustrates the three groups of CEACAM1-related genes in microbats: CEACAMs, PSG I, and PSG II. The species from which the sequences are derived are marked in addition to a three letter species name, by a color/symbol code. While in the genome of *M. lucifugus* (Mlu), *Myotis brandtii* (Mbr) and *Myotis davidii* (Mda) nearly equal numbers of PSGs of group I and group II exit, PSGs from group I dominate in the genome of *E. fuscus* (Efu) and PSG of group II dominate in the genome of *P. parnellii*. Phylogenetic analysis based on amino acid sequences was performed using the MEGA6 software. Numbers on each node indicate the statistical support of bootstrap analysis. Scale bar at the top indicates substitutions per site

We next analyzed CEA families in additional microbat species belonging to different families of Yangochiroptera i.e. *M. davidii*, *M. brandii, E. fuscus* (Vespertilionidae)*, P. parnellii* (Mormoopidae) *and M. natalensis* (Miniopteridae). In all species we found a large number of *CEACAM* genes, most of them again code for secreted PSGs. However, as shown in Fig. 5 only the closely related bat species *M. lucifugus, M. brandii* and *M. davidii* have a large number of PSGs in both PSG subgroups, *E. fuscus* has only two PSGs within the PSG II group but 8 group I members and *P. parnellii* (Mormoopidae) has only PSGs belonging to the PSG II group. In *M. natalensis* (Miniopteridae) only few PSG-like CEACAMs were identified one belonging to the PSG I and four to the PSG II group.

### No PSG-like CEACAMs are found in members of the Yinpterochiroptera suborder

**Fig. 6:**
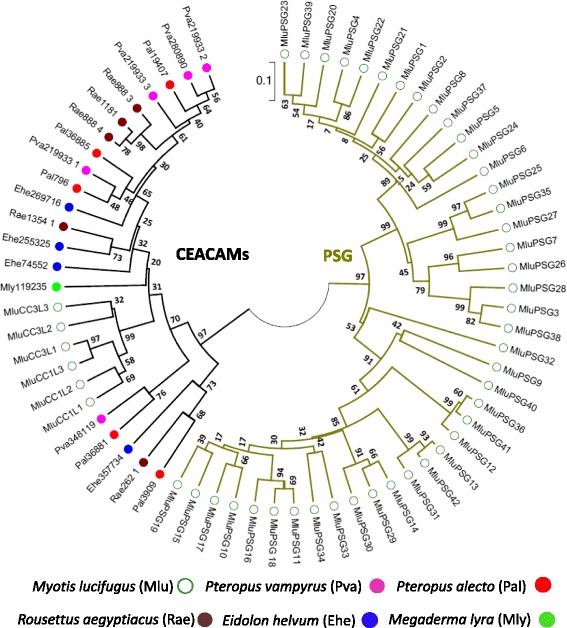
*PSG* genes evolved in Yangochiroptera but not in Yinpterochiroptera bat species. CEACAM1-related IgV-like N domain nucleotides sequences of megabats including *P. vampyrus* (Pva)*, P. alecto* (Pal), *R. aegyptiacus* (Rae) and *E. helvum* (Ehe) and the microbat *Megaderma lyra* (*M. lyra*; Mly) were aligned with CEACAM1-related sequences from *M. lucifugus* (Mlu). Phylogenic analysis was performed using MEGA6. All sequences of megabats and *M. lyra* clustered together with the CEACAM sequences and none with PSG sequences of *M. lucifugus*

To further extend our phylogenetic analyses we analyzed members of the *CEA* gene family of the Yinpterochioptera suborder including megabats (*Pteropus vampyrus* [*P. vampyrus*]*, Pteropus alecto* [*P. alecto*]*, Rousettus aegyptiacus* [*R. aegyptiacus*] and *Eidolon helvum* [*E. helvum*]) as well as members of the microbat families Rhinolophidae and Megadermtidae. Interestingly, in all species of the Yinpterochiroptera suborder all *CEA* gene family members belong to the *CEACAM* subgroup and no *PSG*-like genes could be identified despite the same depth of genomic sequencing of both bat suborders (Fig. 6).

### The ligand-binding domains of PSGs exhibit positive selection

**Fig. 7:**
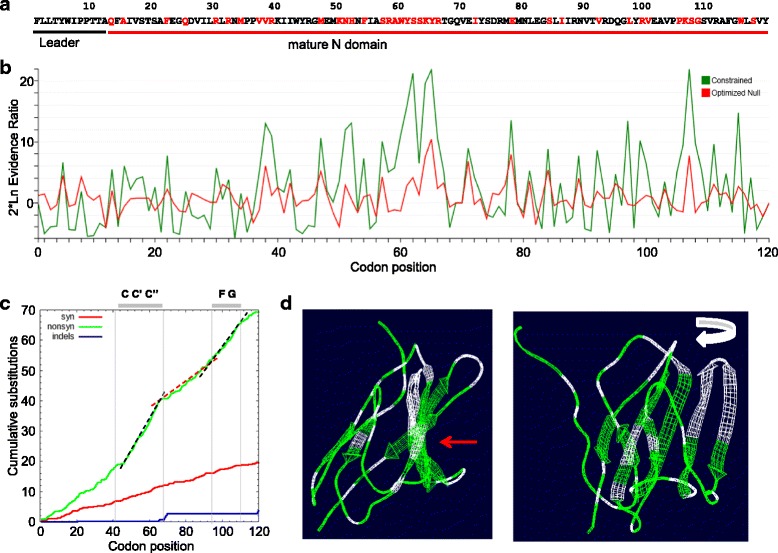
Adaptive evolution of PSGs in Yangochiroptera bat species. **a** For the detection of individual sites under positive selection (red letters) we used MEME software after screening for recombination using GARD software. All PSGs from *M. lucifugus* were used for the analysis. The amino acid sequences encoded by the *M. lucifugus* PSG1 N domain exon is shown with the positively selected amino acids marked in red. **b** Identification of N domain-wide episodic selection. A branch-site unrestricted statistical test for episodic diversification (BUSTED) approach was used for the identification. **c** The accumulation of non-synonymous (*green curves*) and synonymous substitutions (*red curves*) along the N exons of PSGs. The blue curve indicates insertions or deletions of nucleotides. Note preferential accumulation of nonsynonymous mutations in the CC’C″FG β-strand regions (black broken lines) which indicates selection for diversification. This contrasts with a conserved region between CC’C″ and FG β-strands indicated by a red broken line. The location of CC’C″ and FG β-strand regions was determined by 3D modeling (**d**) Three-dimensional modeling (Geno3D) of the N domain of PSG1 from *M. lucifugus*. In the left ribbon model the CC’C″FG β-strands are indicated by an arrow. The right model is horizontally rotated by 90° clockwise. Positively selected sides are shown in green. Note that multiple positively selected sides are located in the CFG face which is known to interact homo- and heterotypically in other CEACAMs

Rapid expansion of gene families is due to selection for a higher gene dosage or for functional diversification of the gene repertoire. Selection for diversification is accompanied by adaptive evolution, i.e. a high ratio of nonsynonymous to synonymous nucleotide substitutions is observed. Therefore, we analyzed the evolution of the presumed ligand-binding domains (IgV-like domains) of bat PSGs using the SLAC and the BUSTED software (see “Material and Methods” section). A mean ratio of nonsynonymous mutations per nonsynonymous site and synonymous mutations per synonymous site (dN/dS) of 1.66 was indicative for positive selection on the IgV-like domains of bat PSGs, which was confirmed by the detection of evidence of episodic diversifying selection using the *likelihood ratio test* (LRT) in the BUSTED software package (*p*-value <0.001). We further determined the selection pressure at individual sites using BUSTED and MEME software. It turned out that multiple amino acid positions are under positive selection indicating that the ligand binding domain is selected for diversification (Fig. 7a-c). Interestingly, most of the amino acids under positive selection are located at the molecular surface of the IgV-like domains as determined by three-dimensional modeling (Fig. 7d).

### Sites under positive selection differ between PSG I, PSG II and CEACAM N domains

**Fig. 8:**
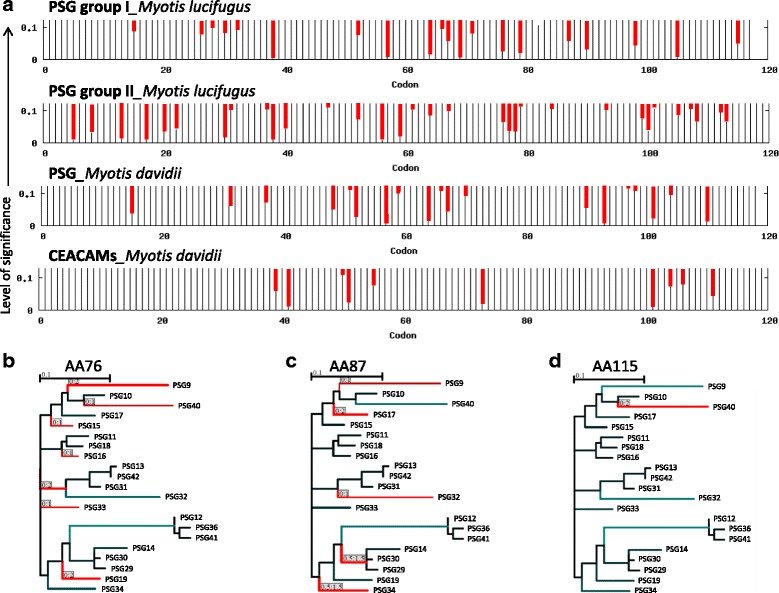
Regions of positive selection differ between PSGs and CEACAMs. **a** Sites within N domain exons (x-axis) with episodic diversifying selection as detected by MEME were plotted (red bars) against the p value (level of significance; y-axis). Genes used for the analyses are indicated. Note that in CEACAMs, PSGs group I and PSGs group II different sides are under positive selection. **b**, **c**, **d** Selection at defined sites of PSGs of group I from *M. lucifugus*. The position of the analyzed amino acid (AA) is indicated on top of the phylogenetic tree. Phylogenetic trees of group I PSGs are shown. Red lines indicate positive selection, black lines neutral selection and blue lines negative selection. Note that at several nodes one arm is under positive selection while the second is under neutral or negative selection. The number of PSGs of which a certain amino acid is under positive selection varies between individual positions. Scale bar at the top indicates substitutions per site

We used MEME and BUSTED software to analyze individual sites under positive selection in order to find regions which may be of functional significance. N domain sequences of all PSGs (*M. davidii*), subgroup PSG I (*M. lucifugus*) subgroup PSG II (*M. lucifugus*) and CEACAMs (*M. davidii*) were aligned separately using “Muscle” (codon alignment) and MEGA6. Alignments were analyzed for N exon-wide episodic selection using BUSTED. In all groups evidence for positive selection was found. We further compared sites of positive selection with a level of significance <0.1 (default parameter BUSTED) (Fig. 8a) of PSG I and PSG II of *M. lucifugus* and noticed that positive selection occurs at different sites in both PSG groups (Fig. 8a). In addition sites of positive selection of PSGs differ from positively selected sites in CEACAM N domains (Fig. 8a). As assumed for a group of diverging genes, individual sites were under different modes of selection in different *PSG* genes (Fig. 8b–d).

### Bat PSGs have a restricted expression pattern

Variation of the ligand-binding domains may be interpreted as a characteristic feature of decoy receptors for pathogen adhesins, therefore, we wondered if secreted PSGs are expressed in the placenta or by immune cells. Unfortunately, we were not able to get placental tissue from microbats. Therefore, we focused our expression analyses on immune cells and determined the transcriptome of lymphoid tissues. Total RNA was isolated from the spleen, thymus, intestine and lymph nodes of one individual of the species *Myotis myotis* which is very closely related to *Myotis* davidii [20] and analyzed by RNA sequencing. All reads related to genes of the *CEA* gene family were analyzed. Remarkably only sequences belonging to the *CEACAM*-related subgroup could be found and none of the expressed genes belonged to the *PSG* group (Table 1).

**Table 1:** *Myotis myotis* CEACAM mRNAs identified by RNA sequencing

Gene	Depth	reads	length^a^
CEACAM18	2.776.660	5799	1877
CEACAM1-like_1	742.742	307	372
CEACAM1-like_2	403.448	143	319
CEACAM1-like_3	148.130	66	401
CEACAM1-like_4	85.574	29	305
CEACAM3-like_1	400.223	661	1480
CEACAM3-like_2	147.817	79	481
CEACAM-like_1	1.430.570	1729	1087
CEACAM-like_2	1.185.171	1390	1054
CEACAM-like_3	96.854	65	604
CEACAM-like_4	222.078	76	308
CEACAM-like_5	126.000	35	250

^a^sequences may be only partial mRNAs

## Discussion

In most species of the superorder Laurasiatheria, the *CEA* gene family is relatively small [11]. However, a few exceptions of this rule exist [11]. Recently, we have identified an expanded *CEA* gene family in the horse the expansion of which is due to the amplification of genes coding for secreted PSG-like CEACAMs [21]. In addition, there are reports indicating that the *CEA* gene family has been expanded in certain microbats [22], namely in *Myotis lucifugus* [11] and in *Myotis davidii* [12]. Such a co-expansion of a gene family in otherwise distantly related species may point to similar selective pressures working on these species during evolution and thereby may provide clues to the function of the gene family. In order to get a comprehensive knowledge of the *CEA* gene family in bats we have analyzed the *CEA* gene family in 12 different bat species. According to the phylogenetic tree based on the sequences of orthologous CEACAMs six of these species belong to the Yangochiroptera and six to the Yinpterochiroptera suborders which is in accordance with phylogenetic trees previously reported by other authors [19, 23]. The maximal number of CEA family-related N domains containing an open reading frame in a single species of Yinpterochiroptera suborder was six. In contrast in Yangochiroptera in particular in the Myotis genus up to tenfold as many CEA family member N domains were found. According to the currently proposed phylogeny of Yangochiroptera Miniopteridae are more closely related to Vespertilionidae than to Mormoopidae [19, 24]. Therefore, it is surprising that we found more *CEA gene family* members in *P. parnellii* than in *M. natalensis*. The most plausible explanation is that the expansion of the *CEA gene family* occurred in both linages independently. Furthermore the difference in the size of the *CEA* gene family in *Myotis* and *Eptesicus* indicates that a second boost of *CEA* gene family expansion occurred between 20 and 25 Mya years ago in the Vespertilionidae [19, 24].

Multiple *CEACAM* genes were found to code for transmembrane proteins with signaling capacities through ITIMs and ITAMs in the cytoplasmic tails. Interestingly, amplification of both gene types coding for ITAM- and ITIM-containing CEACAMs occurred. In the *CEA* gene families of other mammals described previously, a preferential expansion of either genes coding for ITAM-containing proteins (dog) or for ITIM-containing proteins occurred (mouse, horse and, opossum) [9, 11]. Interestingly, a balanced expansion of transmembrane CEACAMs could be found in both Yangochiroptera and Yinpterochiroptera. This may be explained by the fact that multiple ITIM- and ITAM-containing signaling CEACAMs were already present in the last common ancestor of Yangochiroptera and Yinpterochiroptera bat suborders. In the pooled tissues including spleen, thymus, lymph node and intestine we found strong mRNA expression of these signaling CEACAMs, consistent with the view that these CEACAMs are important for immune function. We have previously reported that pairs of ITIM- and ITAM-containing CEACAMs with very similar ligand-binding domains exist in most mammals and even in amphibians [11, 25]. The most plausible explanation for the evolution of these paired receptors is that activation receptors evolved as a countermeasure to the use of the inhibitory receptor by pathogens as cellular receptors. One indication for such a mechanism is that the ligand-binding domain is most similar between ITIM-containing CEACAMs and an ITAM-containing CEACAM which is the case for CEACAMs of *Myotis lucifugus* (CEACAM1L1/CEACAM1L2 and CEACAM3L1) (Fig. 4).

However, the enormous expansion of the *CEA* gene family in certain bat species is due to the amplification of genes coding for a single IgV-like domain followed by an IgC-like domain. The IgC-like domains are of the A2 type (named according to the most similar IgC-like domain of CEACAM1) and are encoded by exons which have either stop codons (at the beginning of the exon) or mutated splice donor sites. The mutation of the splice donor site creates a stop codon which is followed in near proximity by a polyadenylation signal. The presence of a leader sequence indicates that these molecules are secreted. Indeed, this is besides expression by trophoblast cells (which could not be demonstrated directly due to lack of bat placental tissues), the most important classification criterion for PSGs. The closer relationship of the ligand-binding domains (IgV-like domain) between PSGs than to other CEACAMs in a given species represents an additional criterion. Indeed this is the case for bat PSGs. Remarkably, the structure of bat PSGs is very similar to the PSGs recently found in the horse [21] suggesting that both have a common ancestor. Indeed the phylogenetic relationship of bats within Laurasiatheria is still a matter of debate, however several lines of evidence point to a close relationship of bats and horses [23]. For example, Zhang and colleagues used 2492 nuclear-encoded genes to perform maximum-likelihood and Bayesian phylogenomic analysis. Their results vigorously supported bats as a member of Pegasoferae (Chiroptera + Perissodactyla + Carnivora), with the bat lineage diverging from the Equus (horse) lineage ~88 million years ago [12]. Similar findings were obtained on transcriptome level by Papenfuss and coworkers [26].

Interestingly, phylogenetic analysis indicated that two main groups of PSGs exist in bats. Both groups contain an almost equal number of PSGs in Vespertilionidae. In the more distantly related *Pteronotus parnellii* PSGs of subgroup II expanded preferentially (Fig. 5). Taken together our data imply that the PSG group II is the more primordial PSG group being present in all analyzed representatives of the Yangochiroptera. The ancestor of PSGs group I may have arisen in a common ancestor of the Miniopteridae and the Vespertilionidae.

What is the reason for the selective expansion of the PSGs in certain families of Yangochiroptera? Until now PSGs are only described in species having a hemochorial placenta [11]. This is the case for all bat species with PSGs. However, hemochorial placentae are also common within the Yinpterochiroptera suborder; this may suggest that a second prerequisite is needed to lead to PSG expansion. This view is further supported by our previous observation that PSGs did not evolve in hedgehogs, which have also a hemochorial placenta [11]. The most obvious prerequisite is that a primordial PSG is created by duplication of a *CEA* gene family member. This would be a random event with a limited frequency, only occurring in restricted number of mammals with a hemochorial placenta. A second possibility is that a hemochorial placenta is not sufficient to drive PSG evolution but additional specific features of the hemochorial placenta, like special blood flow conditions, invasion depth or immunological challenges are necessary. Indeed, it is well known that placentation of bats is extremely diverse and therefore even placentae with a hemochorial interface may differ considerably [27, 28].

On the other hand the very recent and massive expansion of PSGs makes maternal-fetal communication as the only driving force for PSG evolution in microbats questionable. In particular, positive selection point to an interaction with fast evolving ligands. Ligands fulfilling such requirements are for example pathogen receptors. Indeed several pathogens were described to bind to certain CEACAMs. In humans a variety of bacterial pathogens were identified that bind to various human CEACAMs [29–35]. Furthermore, mouse hepatitis virus, which belongs to the corona viridae group 2 uses CEACAM1 as a cellular receptor to infect susceptible hosts [36, 37]. Bats are known to be prominent reservoirs for corona viruses and therefore it is worthwhile to speculate that in microbats viruses exist or have existed that interact with bat CEACAMs. Indeed, recently a bat corona virus of group 2 was isolated from the common vampire bat *Desmodus rotundus* [38]. Secreted proteins with some similarities to these CEACAMs may function as decoy receptors and thereby limit virus binding to their cellular receptor. Such an interpretation would be consistent with a rapid expansion and a positive selection of the decoy receptors. This hypothesis is even more exciting for secreted proteins at the maternal-fetal interface, which could be involved in the prevention of transplacental infection. We further speculate that such a mechanism of innate immunity may be especially beneficial for an order of mammals that live in large colonies with synchronized pregnancies and an extraordinary close contact to other individuals. The rapid expansion of PSGs in certain bat species together with selection for diversification suggest that bat PSGs could be part of a pathogen defense system by serving as decoy receptors and/or regulators of feto-maternal interactions.

## Conclusions

PSGs are a subgroup of the CEA family. We and others have suggested that maternal-fetal interactions are the drivers of the expansion of PSGs in some mammalian species, including humans and rodents. Both higher primates and rodents have a hemochorial placenta type and the close contact of semi- allogeneic fetal cells with the maternal immune system seems to be responsible for the expansion of PSGs. However, in numerous species although having a hemochorial placenta no expansion of PSGs is observed, arguing against a sole reason of maternal fetal communication for the expansion of PSGs. Our analyses of the *CEA* gene family in bats suggest that the expansion of PSGs could also be pathogen-driven. Therefore, we favor the hypothesis that a hemochorial placenta is a prerequisite for the expansion of PSGs but additional conditions are needed, for example a continuous threat by pathogens, to initiate PSG expansion. The identification of bat PSGs opens now the possibility to further determine the tissue of bat PSG expression as well as the screening for pathogens that bind to PSGs. Future investigations are warrant to test if PSGs play a role in preventing trans-placental infections.

## Methods

### Data sets and nomenclature of genes

Sequence similarity searches were performed using the NCBI BLAST tools “blastn” http://blast.ncbi.nlm.nih.gov/Blast.cgi and Ensembl BLAST/BLAT search programs http://www.ensembl.org/Multi/Tools/Blast?db=core using default parameters. For identification of bat *CEACAM* exons, exon and cDNA sequences from known *CEACAM* and *PSG* genes were used to search “whole-genome shotgun contigs (wgs)” databases limited to organism “Chiroptera (taxid:9397)”. Hits were considered to be significant if the E-value was < e-10 and the query cover was >50%. Once a wgs contig was identified that contained CEACAM-related sequences we confirmed manually the presence of the complete exon by the number of nucleotides and identification of *CEACAM*-typical splice site sequences. Only sequences which were considered to be complete exons were used for further analyses. In a second step we used the identified exon sequences to search the database limited to this bat species in order to identify all existing paralogous *CEACAM* genes. In some species we performed several rounds of searches using sequences of distantly related CEACAMs in a given species. Once we had identified individual exons we predicted the gene structure according to known CEACAMs. The location of different exons on the same contig was a prerequisite for considering that these exons belong to the same gene. Gene predictions were further supported by the identification of “expressed sequence tags (est)” and or predictions in genome builds at NCBI and Ensemble, if available. Short exons, like exons coding for the cytoplasmic tail, were identified by alignments of downstream sequences of identified transmembrane exons with cytoplasmic exon sequences of human CEACAMs. Sequence alignments for exon identification was performed using clustalw (http://www.genome.jp/tools/clustalw/). The following wgs data sets were used: *Myotis lucifugus* AAPE02 (Genome Coverage (GC): 7×; Sequencing Technology (ST): Sanger); *Myotis brandtii* ANKR01 (GC: 120×; ST: Illumina HiSeq 2000); *Myotis davidii* ALWT01 (GC: 110×; ST: Illumina HighSeq 2000); *Eptesicus fuscus* ALEH01 (GC: 84×; ST: Illumina Hi-Seq); *Pteropus vampyrus* ABRP02 (GC: 188×; ST: Illumina); *Pteropus alecto* ALWS01 (GC: 110×; ST: Illumina HighSeq 2000); *Pteronotus parnellii* AWGZ01 (GC: 17×; ST: Illumina HiSeq); *Rhinolophus ferrumequinum* AWHA01 (GC: 17×; ST: Illumina HiSeq); *Megaderma lyra* AWHB01 (GC: 18×; ST: Illumina HiSeq); *Eidolon helvum* AWHC01 (GC: 18×; ST: Illumina HiSeq); *Miniopterus natalensis* LDJU01 (GC: 77×; ST: Illumina HiSeq); *Rousettus aegyptiacus* LOCP02 (GC: 169.2×; Illumina HiSeq; PacBio).

The *CEA* gene family in bats is not well annotated; therefore, we adopted the nomenclature according to the one previously used for the *CEA* gene family of other mammals [11]. Gene names and corresponding sequences are summarized in (Additional file 1).

### Phylogenetic analyses

Phylogenetic analyses based on nucleotide and amino acid sequences were conducted using MEGA5 and MEGA6. Sequence alignments were performed using “Muscle”. The maximum likelihood (ML) method with bootstrap testing (500 replicates) was applied for the construction of phylogenetic trees. To determine the selective pressure on the maintenance of the nucleotide sequences, the number of nonsynonymous nucleotide substitution per nonsynonymous site (dN) and the number of synonymous substitutions per synonymous site (dS) were determined for N domain exons. The dN/dS ratios were calculated after manual editing of sequence gaps or insertions guided by the amino acid sequences for all branches of the resulting phylogenetic trees using the Datamonkey web interface. The mean dN/dS ratios were calculated using the single likelihood ancestor counting (SLAC) algorithm. The synonymous nonsynonymous analysis program (SNAP; http://www.hiv.lanl.gov/content/sequence/SNAP/SNAP.html) allowed the calculation of cumulative average synonymous and nonsynonymous substitutions along coding regions of N domain exons from paralogous and orthologous genes. For the identification of N domain-wide episodic selection we used a branch-site unrestricted statistical test for episodic diversification (BUSTED) approach [39]. For the detection of individual sites under positive selection we used the mixed effects model of evolution software (MEME) [40] after screening for recombination using the genetic algorithm for recombination detection (GARD) software [41].

### Expression analyses

*M. myotis* individuals, which could not survive in nature because of injuries, were used for isolation of tissues. Samples from immune organs including spleen, thymus, intestine and lymph nodes were stored in RNAlater at −80 °C until RNA extraction. Total RNA was extracted and contaminating DNA was removed by DNase I treatment using the RNeasy Mini Kit (Qiagen, Germany). The RNA concentration was determined with the NanoDrop 2000/2000c spectrophotometer (Thermo Fisher Scientific, USA), and RNA integrity was tested by measurement of the 28S/18S rRNA ratio using bioanalyzer Agilent2100 (Agilent Technologies Inc., USA). RNA from spleen, thymus, intestine and lymph nodes were pooled in a mass ratio of 1:1:1:1 and used for de novo transcriptome sequencing by Illumina Hi-SeqTM2000. RNA processing, cDNA library construction, sequencing and data processing were performed in the Beijing Genomics Institute (BGI), Shenzhen, China. For de novo assembly, raw reads were first filtered to remove adaptor sequences and reads with more than 5% unknown bases (N) and more than 20% low quality bases (bases with quality value ≤10), and then clean data were assembled using the short reads assembling program Trinity into non-redundant unigenes [42]. Next, all of the unigenes were annotated by the best hits out of BLASTX alignments against protein databases of non-redundant proteins (NR) (http://www.ncbi.nlm.nih.gov), Swiss-Prot protein (http://www.uniprot.org/uniprot/), Kyoto Encyclopedia of Genes and Genomes (KEGG) pathway (http://www.genome.jp/kegg) and Cluster of Orthologous Groups (COG) (http://www.ncbi.nlm.nih.gov/COG) (E value <0.00001). Those without any match in above databases were further aligned by blastn to nucleotide databases (NT) (E value <0.00001). With NR annotations, GO functional annotations and classifications were obtained using the Blast2GO program [43] and the WEGO software [44], respectively.

## Additional file

Additional file 1: Nucleotide sequences from the N domains of Myotis lucifugus CEACAMs. (DOCX 30 kb)
