# Supplementary material for: Recent expansion and adaptive evolution of the carcinoembryonic antigen family in bats of the Yangochiroptera subgroup
Source: BMC Genomics. 2017 Sep 11;18:717. doi: 10.1186/s12864-017-4106-7 (PMC5594555; doi:10.1186/s12864-017-4106-7)
Supplement: Additional file 1: — Nucleotide sequences from the N domains of Myotis lucifugus CEACAMs. (DOCX 30 kb) [file 12864_2017_4106_MOESM1_ESM.docx]

>Mlu_PSGps1

TCTTCCTCTTAACCTTGTGGTTCTCACCCATCACTGCACAATTTGCTATTGTCTTGACCATAGCTCTTGAAAGGGAGGATGTGATTCTTCGTCTTCCCAATATGCCTCCCGATGTTACAGGGTTTATTTGGTACAGGGGAATAGAGATGAAGTACCATAATTTCATTGGGTCTCTTGCATAGTATTACAGTGTATATCTAAAAGGTCCCGAATACAGTGGTCAAGAGGAAATAAACCATGAGGATCCCTGATCATAAGAAACGTCACTGTGTGGGACCTAGGAATTTACGTCATAGTAGCCGTCCTTCCAAATTCAGGAAGAGAAATAGGATTTGGACAGCTCAATGTCTTCCATGA

>Mlu_PSGps2

TCTTCCTCTTAACCTTCTGGATCCCGCCCACCACTGCACGATTTGGTATTGTCTTGACCATTGCTCTTGAAGGGCAGGATGTGATTCTACGTACCCAAAATAGGCCTCCAGGTGTTACAGGCTTTGTTTGGTACAGGGGAAAAGAGATGAACTACTATACTCTTATTGCATCTCTTACATTGCATTCAAGGCGATTGCTAAGAGGGCCCGAATACAGAGGTCGAGAGACAGTAAATCTTGATGGATCCCTGACCATAAGAAAAGTCACTGTGAGGGACCTAGGAATGTACATCTTAGTAGCCGTCCTTCAAAATTTATAAAAAGTAATTGGATTTGGACAGCTCAAAGTATACC

>Mlu_PSGps3

TCTTCCTCTTAACCTTCTGGATCCCGCCCACCACTGCGCAATTTGCTATTGTCTCGATTAATGCTGCTGAAGGGCAGGATGTGATTCTTCGTACCCACAATAGGCCTCCCAGTTGTGCAGGCTTTATTTGGTACAGGGGAGAAAAGATGGACTACTATCATTTTATTGCATCTGTTGCATGGCATGTGAGATGATATAGAACAGGGCAGGAATACAGTGGTCGAAAGACAGCAAATCTTGAAGGATCCCTGATCATAAGAAAAGTCACTCTGAAGGACACATGAACCTACACCGTAATAGCCATCCTTCAAAATTCACTAAGAGAAATAGGATTTGGACAGCTCAATGTATACC

>Mlu_PSGps4

TCTTCCTCTTGTCCTTGTGGATCCCGCCCACCACTGCACAATTTGCTATTCTCTCCACCATTGCTCTTGAAGGGCAGGATGTGATTCTATGTCTCCGCAATCCACCTCCCGATGTTACAGGGTTTATTTGGTACAGAGGAAGAGAGATGAAGTACCATAATTTCACTGGGTCTCGTGCATGGTATTCCAGTGAATATCTAACAGTTCCCAAATAAAGTGGTCGAGAGGAAATAAACCTTGAAGGATCCCTGATCATAAGAAACGTCACTGTGTGGGACCTAGGAATTTACGTCCTAGTAGCCGTCCTTCCAAATTCACGAAGAGAAATAGGTTTTGGACAGCTCATTGTATTCC

>Mlu_PSGps5

TCTTCCTCTTAACCTTCTGGATCCCACCCACCACTGCACGATTTGGTATTGTCTTGACCAGTGTTCTTGAAGGGCAAGTTGTGATTCGACGTACCCAAAATAGGCCTCCAGGTGTTACAGGCTTTGTTTGGTATAGGGGAATAGAGATGAACTACTATACTCTTATTGCATCTCTTACATTGCATTCAAGGCGATCGCTAAGAGGGCCCGAATACAGTGGTCGAGAGACAGTAAATCTTGATGGATCCCTGACCATAAGAAAAGTCACTGTGTGGGACCTAGGAATGTACATCGTGGTAGCCGTCCTTCAAACTTTACAAAAAGAAATTGGATTTGGACGGCTCAATGTATACT

>Mlu_PSGps6

TCTTCCTCTTAACCTTCTGGATCCTGCCCACCACTGCATGATTTGGTATTGTCTTGACCAGTGCTCTTGAAGGGCAAGATATGATTCTACGTACCCAAAATAGGCCTCCAGGTGTTACAGGCTTTGTTTGGTACAGGGGAATAGAGATGAACTACTATACTCTTATTGCATCTCTTACATTGCATTCAAGGCGATCGCTAAGAGGGTCTGAATACAGTGGTTGAGAGACAGTAAATCTTGATGGATCCCTGACATTAAGAAGTTACTGTGTGGGACCCAGGAATGTACATCGTAGTAGCCGTCCTTCAAAATTTACAAAAAGAAATTGGATTTGGACAGCTCAACGTATACC

>Mlu_PSGps7

TCTTCCTCTTAACCTTCTGGATTCCACCCACAACTGCGCGATTTGCTATTGTCTCGACAAGAGCTGTTGAAGGGCAGTAAGTGATTCTCCGTACCCACAATATGCCTCCTGGTGTTACAGGCTTTATTTGGTACAGGGGAATAGACATGAAGTACTATAATTTAATTGGGATGCTTGCATGGCAGTTTAGTCGATATCTAACAGGTCCGAAATACAGTGGACGAGAGAAAATAAACCTTGAAGGATCTCTGATCATAAGAAACGTCACTGTGAGGGACCTAGGAATCTACATCGTAGTAGCCATCCTTCCAAAGTCACCAAGAGAAATAGGATTTGGACGGCTCAATGTATACT

>Mlu_PSGps8

TCTTCCTCTTAACCTTGTGGATCCCCACCACCACTGCACAATTTGCTATTGACTCGACCATTGCTCTTGAAGGGCAGGATGTGATTCTACGTCTCCGCAATACTCCTCCCAATGTTACAGGGATTATTTGGTACAGGGGAATAGAGATGAAGTACCATAATTTCATTGGGTCTCGTGCATGGAATTACAGTGAATATCTAACAGGTCCCGAATACAGTGGTTGAGAGGAAATAAATCTTGAAGGATCCCTGATCATAAGAAATGTCACTGTGAGGGACCAAGGAGTTTACATCGTAGTAGCCATCCTTCCAAAGTCACCAAGTGAAATAGGATTTGGATGGCTCAGAGTATATC

>Mlu_PSGps9

TCTTCCACTTAACCTTCTGGATCCCACCCAGCACTGCGCGATTTGCTATTGTCTCGATTAATGCTGCTGAAGGGCAGGATGTGATCCTTTGTAACCACAATAGGCTTCCCAGCTGTGCAGGGTTTATTTGGTACAGGGGAGAAAAGATGGACTACTATCATTTTATTGCGTCTGTTGCATGGCATGTGAGATGATATAGAACAGGGCCCAAATACAGTGGTCCAGAGACAGCAAATCTTGAAGGATCCCTGATCATAAGAAAAGTCACTCTGAAGGACACAAGAACCTACACCGTAATAGCCGTCCTTCAAAATTCACTAAGAGAAATAGGATTTGGACGGTTGAATGTATACC

>Mlu_PSGps10

GCTTCCTCTTAACCTTCTGGATCCCGCCCACCACTGCACGATTTGGTATTGTCTTGACCAGTGCTCTTGAAGGGAAAGATGTGATTCTACGTACCCAAAATAGGCCTCCCGGTGTTACAGGCTTTGTTTGGTACAGGGGAATAGAGATGAACTACTATACTCTTATTGCATCTCTTACATTGCATTCAAGGTGATCGCTAAGAGAGCCCGGATACAGTGGTCGAGAGACAGTAAACATTGGTGGATCCCTGACCATAAGAAAAGTCACTGTGTGGGACTTAGGAATGTACATCATAGTAGCCTTCCTTCAAAATTTTCAAAAAGAAATTGAATTTGGACTGCTCAGTGTATACC

>Mlu_PSGps11

TCTTCCTCTTATCCATGTGGATCCTGCCCACCGTTGCCCTATTTTCTTTTGTCTCAACCAATGCTGCTGAAGGGCAGGATGTGATTCTACATATCTGCAATAGGCTTCTCTCTGTTGCAGGGTTTATTTGGTACAGGGGAATAGTGATGAAGCACTATTATTTAATTGGGTCTATTGCATGGCATTTAAGTCGATTTATTACAGGGCCCAAATACAGTGGTCGAGAGACAGCAAACCTTGAAGGATCTCTGATCATAAGAAATGTCACTGTGAGGGACATAGGAATCTATACCGTAGAAGCCCTCGTTCTAAATTCACAAAGTGTAATAGGATTTGGCTGGCTCAGTGTATACT

>Mlu_PSGps12

TCTTCCTCTTAACCTTCTGAATCCCATCCACCACTGCACGATATGGTATTGTCTTGTCCAGTGTTTTTGAAGGGCAAGTTGTGATTCTACGTACCAAAAATAGGCCTCCAGGTGTTACAGGCTTTGTTTGGTACAGGAAAAGAGATGAACTACTATATTCTTATTGCATCTCTTACATTGCATTCAAGGCGATCGCAAAGAGGGCCGGAATACAGTGGTCAAGAGACAGTAAAACTTGATGGATCCCTGACCATAAGAAAAGTCACTGTGTGGGACTTAGGAATGTACATTGTAGTAGCCGTCCTTCAAAATTTACAAAAAGAAATTGGATTTGGACAGCTCAATGTATACT

>Mlu_PSGps13

TCTTCCTCTTAACCTTGTGGATCCCGCCCACCACTGCAGAATTTGATACTAAATGGAACTTTGCTCTTGAAGGGCAGGATGTGATTCTACGTCTTCGCAAAACGCCTCCCGATGTTATAGGGTTTATGTGGTACAGGGGAAAAGAGATGGAGTACCATAATTTCATTGGGTCTCTTGCAAGGGATTCCAATGAATATCTCACAGGTCCGGAATACAGTGGTTGAGAGGAAATAAACCTTTAAGGATCCCTGATCATAAGAAAAGTCACTGTGAGGGACCAAGGAGTTTACATCATAGTAGCCATCCTTCCAAAGTCACGAAGAGTAAGAGGATTTGGATGGCTCAGAGTATACA

>Mlu_PSGps14

TCTTCCTCTTAACTCTTTGAATCCCGCCCACCACTGCGCGATTTGCTGTTGTCTCGACCAGTGTTCTTGAAGGGCAGGATGTGATTCTACATACCCGCAATAGGCCTCCCAGTTGTGCAGGCTTTATGTGGTACAGGGGTGAAAGGACTAACTCCAAAAATTTGATTATGTATGTAACTCGTATTCCAGTGAATATCTAACAGGTCCAAAATACAGTGGTCGAGAGGAAATAACCATTGGAGGACACCTGATCATAAAAAACGTCACCCTGAAGGACACAGGAATGTACATGGTAGTAGCCATCCTTCGAAATTTAAAAATAGAAGCAGGATTTGGACGGCTCAGAGTATACC

>Mlu_PSGps15

TCTTCCTCTTAACCTTGTGGATCCCCACCACCACTGCACAATTTGCTATTGACTCGACCATTGCTCTTGAAGGGCAGGATGTGAGTCTACGTCTCAGCAATAAGCCTCCCGATGTTACAGGGTTTATTTGGTACAGGGGAATAGGACTAACTCCAAAAATTTGATTATGTATGTAACTCATTTAAGACGATATAGAACAGGGTCTGCATACAGTGGTCGAGAGGAAATAAACCTTGAAGAATCCCTGATCATAAGAAACATCACTGTGTGGGACCTAGGAATTTGCGTCATACTAGCTGTCCTTCCAAATTCACGAAGAGAAATGGGATTTGGAAAGTGCAATGTATTCCGGGA

>Mlu_PSGps16

TTTCACTCTTAAATTTTGGAGCCTGCCCACCACTGCCCAACTCCCTATTGTGTCAACCAATGATGCCGAAGGGAAGGATGTGATTCTATGTATCCGCAATAATCCTCCCAATGCTATAGGCTTCATGTGGTACCGGGGGGAAAGGGGCAAACTACTATCATCATATTGCAACTCTCACAACACAGATAAGAATTTATAAAACAGGGTATGCATATAGCGGTCGAGAGAAAATAAACTATGATGGATCCTTGCAATTAATGCAGGTCACCCAGAAGGATACTGGAATCTACACCATAATAATCTACCTTCCAGGCTCAATAAAAGAAATAGGATTTGGATTACTCAATGTATATG

>Mlu_PSGps17

TTTCACTCTTAAATTTCTGGAGCCTGCCCACCACTGCCCAACTGGCTATTGTGTCAACCAGTGCTACCGAAGGACAGGATGTGATTCTACGTATCCGCAATATGCCTCCCCATGTAAGAGGCTTCATGTGGTACAGGGGGGGAAGATCCAAACTATGATTATAATATTGCACATCTTGGAATGTTGCCAGGACATAGAGCAGGGCCTGCACACAGCAGTCGAGAGCAAATAAATTTTGATGGATCCTTGCTGATAAAGAGTGTCACCCTGAATGACACAGGAATCTACAGCATAGTAGTCTAACTTTGAGGCTCAAAAAAAGAAATTGGATTTGGACAGCTCAATGTATATG

>Mlu_PSGps18

TTTCACTCGTAAATTTCTGGAGCCTGCGCACCACTGCCCAACTCCCTATTGTGTCAACCAATGCTGCCGAAGGGAAGGATGTGATTCTATGTATCAGCAATAAGCCTCCCAATGCTATAGGCTTCATGTGGTATGGGGGAGGGGGGGAGGGCAAACTACTATCATCATATTGCATCTCTCACAACACACATAAGAATTTAAAAAACAGGGTATACATACAGCATGGATCCTTGCAATTAAAGCAGGTCACCCAGAGGACACAGGGATCTACACCATAGTAGTCTACCTTCCAGGCTCAATAAAAGAAAAAGGATTTTGGACGGCTTAATGTATACG

>Mlu_PSGps19

TTTCACTCTTAAATTTCTGGAGCCTGCCCAGCACTGCCCAACTCACTATTGTGTCAACCAATGCTGCAGTAGGGAAGGATGTGATTCTCCGCATCCGCAATATGCCTCCTGTTGTTACAGGCTTCATGTGGTACAGGGGGAAGGGGCAATCTATGAACATAATATTGCAAATCTTGGAATGTCCTGAAGACGTAGAACAGGGCCTGCATACAGCGGTCGAGAGCAAATAAACTTTGATAGATCCTTGCTGATAAAGAGGGTCACCCAGAAGGACACAGGAATCTACACGGTAGTAGTCTACTTTCCAGACTCAATAAAAGAAATAGGATTTGGAAGGCTCAATGTATACG

>Mlu_PSGps20

TTTCACTCTTAAATTTCTGGATCATGCCCTCCACTGGCCAACTCGCTGTTTTGTCGAAAGGAAGGATGTGATTCTACATATCCGCAATGTGCCTCCCGATGTTAGAGGCTTCATGTGGTACAGGGCGAAAGGGGCAAACTAGAAACATGATATTGCACGTCTTGGAACGTTCCTAAGAAAACATAGAACAGGGCTTGAATACAGCAGTCGAAGGCAAATAAACTTTGATGGATCCTTGCAATTAAAGCAGGTCACCCAGAAGGACACAGGAATCTACATTGTAATAATCTACCTTCCAGGCTCAATAAAAGAAATAGGATTTGCAGGGTTCAATGTATAAGG

>Mlu_PSGps21

TCTTCCTCTTAACCTTGTGGATTCCGCCCACCACTGCACGATTTGAGATTGTCAAGACCAGTGCTCTTGAAGGGCAGGATGTGATTCTACGTACCCAAAATATGCCTCCCAATGTTACAGGCTTTTTTTGGTACAGGGGATTCATGGTGAACTACACTAATTTAATTGTATATGTGTCATGGTGTTCAGGGCAATATATAAAAGGGCCCAAATACACTGGTCGAGAGACAGTAAAATTTGATGGATCTCTGATCATAAGAAACGTCACTGTGTGGGACCTAGGAATCTACATCGGAGTAGCTGTCCTTAACCATTCAAAAACAGAAATAGGATTTGGATGGCTCAATGTATACC

>Mlu_PSGps22

TCTTCCTCTTAACCTTGTGGATTCCGCCCACCACTGCACGATTTGAGATTGTCATGACCAGTGCTCTTGAAGGGCAGGATGTGATACTACGTACCCAAAATAGGCCTCCCGGTGTTACAGGCTTTATTTGGTACAGGGGATTCGTGATGAACTACACTAATTTAATTGCATATGTATCATGGAGTTCAGGGAAATATATAAAAGGGCCCGAATACACTGGTCGAGAGACAGTAAAATTTGATGGAACCCTGACCATAAGAAACGTCACTGTGTGGGACCTAGGAATCTACATCGTAGTAGCCATCCTTCACCATTCAAAAACAGAAATAGGATTTGGACGGCTCAATGTATACC

>Mlu_PSGps23

TCTTCCTCTTAACCTTGTGGATCCCGCCCACCACTGCGCAATTTGCTATTGTCTCGATTAATGCTGCTGAAGGGCAGGATGTGATTCTTCGTACCCGCAATAGGCCTCCCACTTGTGCAGGCTTTATTTGGTACAGGGGAGAAAAGATGGACTACTATCATTTTATTGAGTCTGTTGCATGGGGTGTGAGACGATATAGACAGGGCCCGAATACAGTGGTCGAGAGACAGCAAATCTTGAAGGATCCCTGATCATAAGAAAAGTCACTCTGAAGGACACAGGAACCTACACCGTAATAGCCGTCCTTCAAAATTCACTAAGAGAAATAGGATTTGGACAGCTCAATGTATACCGGGA

>Mlu_PSGps24

CCTCACTCTTAACCTTCTGGAGCCTGCCCACCACTGCCCAGCTCACTATTGAATCAGTGCCGCCCAATGGTGCCGAAGGGAAGGATGTGCTTCTCCGTGTCCACAACCTGCCAGGGTTTCTTGGAGGTTATGTCTGGTTCAAAGGGGAAAGTGTGGACAGCAACCATCAAATTGCATCATATATAATAGATACTCAAGAAATACACCCAGGACTTGCATACAGCGGTCGAGAGACAATATACCCCAACGGGTCCCTGCTGTTCCAGAACGTCACCCTGCAGGATACAGGGTACTACACTCTACTAGCCATAAGGAAAACTTTTCAAGGTGAAATAGTAACCGGACAGCTCCGTGTATACC

>Mlu_PSGps25

TCTTAAATTTCTGGATCGTGCCCTCCACTGGCCAACTCGCTGTTTTGTCAAAAAATACTGCCAAAGGGAAGGATGTGATTCTACATATCCGCAATGTGCCTCCCAATGTTAGAGGCTTCATGTGGTACAGGGGGAAAGGGGCAAACTACAAACATAATATTGCAAGACTTGGAATGTTCCTAGGAAGACATAGAACAGGGTCTGCATACAGCGGTCAAGAGCAAATAAACTTTGATGGATCCTTGTAGTTAAAGAGGGTCACCCAGAAGGACACAGGAATCTACACAGTAGTAGTCTACCTTCCAGACTCAAAAAAAGAAATAAGATTTGGACGGCTCAATGTATATG

>Mlu_PSGps26

TTTCACTCTTAAATTTCTGGAGCCTGCCCACCACTGCCCAACTCGCTATTGTGTCAACCAATGCTGCCGAAGGAAGGATGTGATTCTACGTATCCGCAATAAGCCTCCCAATGCTATAGGCTTCACGTGGTACCGGGGGGAAGGGGCAAACTACTATCATCATATTGCAACTCTCACAACACACATAAGAGTTTATAAAACAGGGCATGCATACAGCGGTCGAGAGCAAATAAACTATGATGGATCCTTGCAATTAAAGCAGGTCACCCAGAAGGACACAGGAATCTACACCGTAGTAGTCTACCTTCCAGGCTCAATAAAAGAAATAGGATTTGGACGGCTCAATGTGTACG

>Mlu_PSGps27

TTTCACTCTTAAATTTATGGAGCCTGCCCACAACTGCCCAACTCGCTATTGTGTCAACCAATGCTGCTGAAAGGAAGGATGTGATTCTACGTATTGCAATATGCCTCCCAATTATATAGGCTTCATGTGATACAGGGGGGAAGGGGCAAACTACTATCACCATATTGCAAGTCTTGCATTGCACATAAGAACTTCTAGAAGAGGGCGTGCATACAGCGGTCAAGAGCAAATAAACTTTGATGGATCCTTCAGTTAAAGAGGGTAACTCAGAAGGACACAGGAATCTACACCCTAGTAGTCTACCTTGGAGGCACAAAAAAAAGCAATAGAATTTGGACGGCTCAATGTATACA

>Mlu_PSGps28

TTTCACTCTTAATTTTCTGGATCGTGCTCTCCACTGGCCAACTCGCTGTTTTGTCAAAAAATACTGCCGAAGGGAAGGATGTGATTCTACGTATCCGTAATGTGCCTCCTGATGTTAGAGGCTTATGTGGTGCAGGGGGAAAGGGGCAAACTACAAACATAATACTGCACGTCTTGGAATGTTCCTAGGAAGACATAGAATAGGTCCTGCATACAGCGGTCGAGAGCAAATAAATTTTGATGGATCCTTGTAGTTAAAGAGGGTCACCCAGAAGGACACAGGACTCTACACCGTAGTAGTCTACCTTCCAGACACCAAAAAAGAAATAAGATTTGGACGGCTCAATGTATATG

>Mlu_PSGps29

TTTCACTCTTAAATTTTGGAGCCTGCCCACCACTGCCCAACTTGCTATTGTGTCAACCAATGCTGCCGAAGGGAAGGATGTGATTCTATGTATCCGCAATAATCCTCCCAATGCTATAGGCTTCATGTGGTACCGGGGGGGAAGGGGCAAACTACTATCATCATATTGCAACTCTCACAACACACATAAGAATTTATAAAACAGGGTATGCATACAGCGGTCGAGAGAAAATAAACTATGATGGATCCTTGCAATTAAAGCAGGTCACCCAGAAGGATACAGGAATCTACACCGTAATAATCTACCTTCCAGGCTCAATAAAAGAAATAGGATTTGGATTACTCAATGTATATG

>Mlu_PSGps30

TTTCACTCTTAAATTTTGGAGCCTGCAACCACTGCCCAACTCGCTATTGTATCAACCAATGCTGCCAAAGGGAAGGATGTGATTCTATGTATCCGCAATATGCCTCCCAATGCTATAGGCTTCATGTGGTACCGGGGGAGAAGGGGCAAACTACTATCATCATATTGCAACTCTCGCAACACACATAAGAATTTATAAAACAGGGTATGCATACAGCGGTCGAGAGAAAATAAACTATGATGGATCCTTGCAATTAAAGCAGGTCACCCAGAAGGACACAGGAATCTACACAGTAATAATATACCTTCCAGGCTCAATAAAAGAAATAGGATTTGGATTACTCAATGTATATCATGA

>Mlu_PSGps31

AATCACTCTTAAATTTCTGGAACGTGCCCTCCACTGGCCAACTTGCTGTTTTGTCAAAAAATACTGCCGAAGGGAGGGATGTGATTCTACATATCCGCAATGTGCCTCCCGATGTTAGAGGCTTCATATGGTACAGGGGGAAAGGGGCAAACTACAAACATAATATTGCACGTCTTGGAATGTTCCTAGGAAGACATAGAACAGGGCCTTCATACAGCAGTCGAGAGCAAATAAATTTTGATGGATCCTTGTAGTTAAAGAGGGTCACCCAGAAGGACACAGGAATCTACACCGTAGTAGTCTACCTTCCAGACTCAAAAAAAGAAATAAGATTTGGATTACTCAATGTATATG

>Mlu_PSGps32

TTTCACTCTTATATTTCTGGAGCCTGCCCACCACTGCCCAACTCGCTATTGTGTCAACCAATGCTGCCAAAGGGAAGGATGTGATTCTAGGTATCCGCAATAAGCCTCCCAATGCAATAGGCTTCTCGTGGTACCGGGGGAAGGGGCAAACTACTATCATCATATTGCAACTCTCAAAACACACATAAGAATTTATAAAACATGGTATGCATACAGTGGTCGAGAGAAAATAAACTATGATGGATCCTTGCTGATAAAGAGGGTCACCCAGAAGGACACAGGAATCTATACCCTAGTAGTCTACCTTCCAGGCTCAATAAAAGAAAAAGGATTTGGGTGGCTCAATGTATACA

>Mlu_PSGps33

TCTTCCTCTTAACCTTCTGGATCCTGCCCACCACTGCACGATTTGGTATTGTCTTGACCAGTGCTCTTGAAGGGCAAGATGTGATTCTACGTACCCAAAATAGGCCTCCAGGTGTTACAGGCTTTGTTTCGTACAGTGGAATAGAGACGAACTACTATACTCTTATTGCATCTCTTACATTGCATTCAAGGCGATCGCTAAGAGGGTCTGAATACAGTGGTCGAGAGACAATAAATCTTGATAGATTTCTGACATTAAGAAGTTACTGTGTGGGACCCAGGAATGTACATCGTAGTAGCCGTCCTTCAAAATTTACAAAAAAAAATTGGATTTGGACAGCTCAACGTATACC

>Mlu_PSGps34

TCTTCCTTTTAACCTTGTGGATCCCGCCCACCACTGCACAATTTGCTATTGTCTCAACCATTGCTCTTGAAGGGCAGGATGTGATTCTACGTCTTCGCAATATGCCTACCGATGTTACAGGGTTTATTTGGTACAGGGGAATAGAGATGAAGTACCATAATTTCATTGGGTCTCTTGCATAGTATTACAGTGTATATCTAACAGGTCCCGAATACAGTGGTCAAGAGGAAATAAACCTTGAAGGATCCCTGATCATAAGAAAGGTCACTGTGTGGGATCTAAGAATTTACGTCGTAGTAGCCTTCCTTCCAAATTCACGAAGAGAAATAGGATTTGGGCAGCTCATTGTCTTCCAGGA

>Mlu_PSGps35

TCTTCCTCTTAACCTTCTGGATCCCGCCCACCACTGCGCGATTTGCTATTGTCTCGATTAATGCTGCTTAAGGGAAGATGTGATTCTTCATACCTGCAATAGGCCTCCCAGTTGTTCAGGCTTTATTTGGTACAGGGGAGACAAGACCGACTACTATCGTTTTATTGCGTCTGTTGCATGGAATGTGAGACGGTATAGAACGGGGCTCGAATACAGTGGTCAAGAGACAGCAAATCTTGAAGGATCCCTGATCATAAGAAAAGTCACTCTGAAGGACTCTGAAACCTACACCATAAAAGCCGTCCTTCAAAATTCACTAAGAGAATTAGGATTTGCATGGCTCAATGTATACCGGGA

>Mlu_PSGps36

TCTTCCTCTTAACCTTGTGGATCCCGCCCACCACTGCGCGATTTGAGATTGTCATGACCAGTGCTCTTGAAGGGCAGGATTGAAGGGCAGGATGTGATACTACGTACCCAAAATAGGCCTCCCGGTGTTACAGGCTTTATTTGGTACAGGGGATTCGTGATGAACTACACTAATTTAATTGCATATGTATCATGGAGTTCAGGGAAATATATAAAAGGGCCCGAATACACTGGTCGAGAGACAGTAAAATTTGATGGAACCCTGACCATAAGAAACGTCACTGTGTGGGACCTAGGAATCTACATCGTAGTAGCCATCCTTCACCATTCAAGAAGAGAAATAGGATTTGGACGGCTCAATGTATACC

>Mlu_PSGps37

TCTTCCTCTTAGCCTTCTGGATCCCGCATGCCACTGCACGATTTGCTGTTGTCCTGCTCTTGAAGGGCAGGATGTGATTCTGCATACCCAAAAGAGGCCTCGTGGTGTTACAGTCTTTAGTTGGTACAGGGGATTAGACTTGAACTACTCTAATTTTATTGGGTCTGATGGATGGCGTTCAAGGCGATATGTAACAGGGCCCGAATACACTGGTCGAGAGAAAGTACAACTTAATGGATCCCAGATGATAAGAAACGTCACTGTATGGGACCTAGGAATCTACATCGTAGTAGCCACCCTTCTAAATGCAGAAAGAGAAATAAGATTTGGACGGCTCACTGTATACCATGA

>Mlu_PSGps38

GCTCACTCTTAACCTTCTGGAGCCTGCCCACCACTGCCCAACTCACTATTGGATCAGTGCCACCCAATGCTGCCAAAGGGAAGGATGTGCTTCTTTGTGTCCACAATCTGCCATGGTTTCTTGGAGGCTATGTCTTGTTTAAAGGGGAAAGTGCAGACAGCAACCATCAAATTGTATCATATGTAATAGATGCTCATGAAATACAACCAGGACCTGCATACAGCAGTCAAGAGACAATATACCCCAATGGATCCCTGCTGTTCCAAAGGTCACCTTGAAAGACACAGGATACTACACCATAGTAGGCTTACTATATGATTCAAAAACAGAAATAGGGCCCAAGCCGGTTTGGCTCA

>Mlu_PSGps39

AATCACTCTTAAATTTCTGGAACGTGCCCTCCACTGGCCAACTCGCTGTTTTGTCAAAAAATACTGCTGAAGGGAGGGATGTGATTCTACATATCCGCAATGTGCCTCCCGATGTTAGAGGCTTCATGTGGTACAGGGGGAAAGGGGCAAACTACAAACATAATATTGCACGTCTTGGAATGTTCCTAGGAAGACATAGAACAGGGCCTTCATACAGCAGTCGAGAGCAAATAAAATTTGATGGATCCTTGCTGTTATAGCGGGTCACCCAGAAGGACACAGGAATCTACACCGTAGTAGTCTACCTTCCAGACTCAAAAAAAGAAATAAGATTTGGACAGCTCAATATATATG

>Mlu_PSGps40

TTTCACTCTTAAATTTCTGGAGAATGCCCACCACTGCCCAACTCGCTATTGTGTCAACCAATGCTGCTGAAGGGAAGGATGTGATTCTACGTATCCGAAATATGCCTCCCGATTGTATAGGTTTCACGTGGTAGGGGGGAAGGGGCAAACTACAGACATAATATTCCAAGTCTTGCATTGCACATAAGAACTTATAGAACAGGGCGTGCATACAGCGGTTGAGAGCAAATAAACTCTGATGGATCCTTATAGTTAAAGAGGGTCACCCTGAAGGAAACAGGAATCTACACCATAGTAGTCTACTTTTGAGGCTCAAAAAAAGAAATAGGATTTGGATAGCTCAATGTATACA

>Mlu_PSGps41

TCTTCCTCTTAACCTTGTGGATCCCGCCCACCACTGTGCGATTTGCTATTGTCTCGATTAATGCTGCTGAAGGGCAGGATGTGATTCTTCGTACCCGCAATAGGCCTCCCACTTGTGCAGGCTTTATTTGGTACAGGGGAGAAAAGATGGACTACTATCATTTTATTGAGTCTGTTGCATGGGGTGTGAGACGATATAGACAGGGCCCGAATACAGTGGTCGAGAGACAGCAAATCTTGAAGGATCCCTGATCATAAGAAAAGTCACTCTGAAGGACACAGGAACCTACACCGTAATAGCCGTCCTTCAAAATTCACTAAGAGAAATAGGATTTGGACAGCTCAATGTATACCGGGA

>Mlu_PSGps42

TCTTCCTGTTAACCTTCTGGATCCTGCCCACCACTGCGGAATTTGAGATGGTCATGACCAGTGCTCTTGAAGGGCAGGATGTGATTCTACGTACCCAAAATAGGCCTCCCAATGTCACAGGCTTTATTTGGTACAGGGGAGAAAAGATGGACTACTATCATTTTATTGAGTCTGTTGCATGGGGTGTGAGACGATATAGACAGGGCCCGAATACAGTGGTCGAGAGACAGTAAAATTTGATGGATCCCTGATCATAAGAAACGTCACTTTGTGGGACCTAGGAATCTACATCGTAGTAGCCATCCTTAACGATTCAGATACAGAAGTAGGATTTGGATGGCTCAGTGTATACC

>Mlu_PSGps43

TCTTCCTCTTAGCCTTCTGGATCCCGCACACCACTTCGTGATTTGCTGTTGTCTCGACCAATGCTCTTGAAGGGCATGATGTGATTCTGCATACCCAAAATAAGCCTCGCGGTGTTACAGTCTTTAGTTGGTACAGGGATTAGACTTGAACTACTCTAATTTTATTGAGTCTGTTGCATGGGGTGTGAGACGATATAGACAGGGCCCGAATACAGTGGTCGAGAGACAGCAAATCTTGAAGGATCCCTGATCATAAGAAACGTCACTGTGTGGGACCTAGGAATCTACATTGTAGTAGCCACCCTTCTAAATGCAGAAAGAGAAATAGGATTTGGACGGCTCAATGTATACC

>Mlu_PSGps44

AATCACTCTTAAATTTCAGGAACGTGCCCTACACTGGCCAACTCGCTGTTTTGTCAAAAAAATACTGCCGAAGGGAAGGATGTGATTCTACATATCCGCAATGTGCCTCCCGATGTTAGAGGCTTCATGTGGAACAGGGGGAAAGGGGAAAACTACAAACATAATATTGCAGGTCTTGGAATGTTCCTAGGAAGACATAGAACAGGGCCTGCATACAGCGGTCGAGAGCAAATAAAATTTGATGGATCCTTGCTGTTAAAGAGGGTCACCCAGAAGGACAAAGGAATCTACACCATAGTAGTCTAACTTCCAGACTCAAAAAAAGAAATACAATTTGGACTGCTCAATATATTTA

>Mlu_PSGps45

TCTTCCTCTTAACCTTGTGGATCCCGCCCACCACTGTGCGATTTGCTATTGTCTCAATTAATGCTGCTGAAGGGCAGGATGTGATTCTTCGTACCCGCAATAGGCCTCCCAATTGTGCAGGCTTTATTTGGTACAGGGGAGAAAAGAAGGACTACTATCATTTTATTGCTTCTGTTGCATGGCATGTGAGACGATATAAAACAGGGCCCGAATACAGTGGTTAGGAGACAGCAAATCTTGAAGGATCCCTGATCATAAGAAAAGTCACTCTGAAGGACACAGGAACCTACACGGTAATAGCCGTCCTTCAAAATTCACTAAGAGAAATAGGATTTGGACTGCTCAATGTATACCATGA

>Mlu_PSGps46

TCTTCCTCTTAACCTTCTGGATCCCGCCCACCACTGCACTATTTGGTATTGTCTTGACCAGTGTTCTTGATGGGCAGGATGAGATTCTACGTACCCAAAATAGGCCTCCAGGTATTACAGGCTTTGTTTGGTATAGGGGAAGAGAGATGAACTACTATACTCTTATTGCATCTCTTACATTGCATTCAAGGTGATCGCTAAGAGGGCCCTAATTCAGTGGTCCAGAGACAGTAAACATTGATGGATCCCTGACCATAAGAAAAGTCACTGTGTGGGACCTAGGAATGTACATTGTAGTAGCCATCCTTCAAAATTTTCAAAAAGAAATTGGATTTGGATGGCTCAATGTATACC

>Mlu_PSGps47

TTTCACTCTTAAATTTCTGGAGCCTGCCCACCGCTGCCCAACTCGCTTTTGTGTCAACCAAGACTACCGAAGGGTAGGATGTGATTCTACTTGTCCGCAATATGCCTCTCAATGCTAGAGGCTTCCTGGGGTACAGGGGGGAAGGGGCAAACTACAAACATAAAATTGCACGTCTTGGATTGTTCCCAGGACATAGAACAGGGCCTGCATACAGAGGTCAAGAGCAAATAAACTTTGATGGATCCTTGCAGTTACAAAGGCTCACCCAGAATGACACAGGAATCTACATAGTACTACTCTACATTCCCAGCATACTAAAAGAAATAGGATTTGGACGGCTCAATGTATATG

>Mlu_PSGps48

TCTTCCTCTTAACCTTCTGGATCCCGCCCACCACTGCACGATTTGGTATTGTCTTGACCAGTGCTCTTGAAGGGCAAGATGTGATTCTACGTACCCAAAATAGGCGTCCAGGTGTTACAGGCTTTGTTTGGTTCAGGGGAATAGAGATGAACTACTATACTCTTATTGCATCTCTTACATTGAATTCAAGGTGATCGCTAAGAGGGCCCGAATACAGTGGTCGAGAGACAGTAAACTTTGATGGATCCCTGACCATAAGAAAAGTCACTGTGTGGGACCTAGGAATGTACATTGTAGTAGCCATACTTCACCATTCAAAAACAGAAATAGGATTTGGATGGCTCAATGTATACT

>Mlu_PSGps49

TTTCACTCGTAAATTTCTGGAGCCTGCGCACCACTGCCCAACTCGCTATTGTGTCAACCAATGCTGCCGAAGGGAAGGATGTGATTCTATGTATCAGCAATAAGCCTCCCAATGCTATAGGCTTCATGTGGTATGGGGGAGGGGGGGGAGGGCAAACTACTATCATCATATTGCATCTCTCACAACACACATAAGAATTTAAAAAACAGGGTATACATACAGCATGCATCCTTGCAATTAAAGCAGGTCACCCAGAGGACACAGGGATCTACACCATAGTAGTCTACCTTCCAGGCTCAATAAAAGAAAAAGGATTTTGGACGGCTTAATGTATACG

>Mlu_PSGps50

TCTTCCTCTTAACCTTGTGGATCCCGCCCACCACTGCAGAATTTGGTATTGTCTAGAAATTTGCTCTTGAAGGGCAGGATGTGATTCTACGTCTCCGCTATACGCCTCCCGATGTTACACGGTTTATTTGGTACAGGGGAATGGAGATGACATACCATAATTTCATTGGGTCTCTTGCATTGTATCCCAGGAAATATCTAACAGGTCCCAAATACTGTGGTCGAGAGGAAATAAACCGTGAAGGATCCCTGATCATAAGAAACGTCACTGTGAGGGACCAAGATATTTATGGCGTAGTAGCCGTCCTTCCAATGTCACGAGGTGCAACAGGATTTGGATGGCTCAGTGTATACA

>Mlu_PSGps51

TCTTCCTCTTAACCTTCTGGATCCCGCCCACCACTGCACTATTTGGTATTGTCTTGACCAGTGTTCTTGATGGGCAGGATGAGATTCTACGTACCCAAAATAGGCCTCCAGGTATTACAGGCTTTGTTTGGTATAGGGGAAGAGAGATGAACTACTATACTCTTATTGCATCTCTTACATTGCATTCAAGGTGATCGCTAAGAGGGCCCTAATTCAGTGGTCCAGAGACAGTAAACATTGATGGATCCCTGACCATAAGAAAAGTCACTGTGTGGGACCTAGGAATGTACATTGTAGTAGCCGTCCTTCAAAATTTTCAAAAAGTAATTGAATTTGGATGGCTCAATGTATACC

>Mlu_PSG1

TCTTCCTCTTAACCTACTGGATCCCGCCCACCACTGCACAATTTGCTATTGTCTCGACCAGTGCATTTGAAGGGCAGGATGTGATTCTAAGACTCCGCAATATGCCTCCCGTTGTTAGAAAGATTATTTGGTACAGGGGAATGGAGATGAAGAACCATAATTTCATTGCATCTCGTGCATGGTATTCCAGTAAATATCGAACAGGTCAAGTGGAAATATACAGTGATCGAATGGAAATGAACCTTGAGGGATCCCTGATCATAAGAAACGTCACTGTGAGGGACCAAGGACTTTACCGCGTAGAAGCCGTCCCTCCAAAGTCAGGAAGTGTAAGAGCATTTGGATGGCTCAGTGTATACC

>Mlu_PSG2

TCTTCCTCTTAACCTACTGGATCCCGCCCACCACTGCTGGATTTGCTATTGTCTCGACCATTGCATTTGAAGGGGAGGATGTGATTCTAAGTCTCCGCAATATGCCTCCCGATGTTATAAAGATTATTTGGTACAGGGAAATAGAGATGAAGTGCCATAATTTCATTGCATCTCGTGCATGGTATTCCAGTGAATATCTAACAGGTCCCAAATACAGTGGTCGAGAGGAAATAAACCTTGACGGATCCCTGATCATAAGAAACGTCACTGTGAGGGACCAAGGAGTTTACATCGTAGTAGCCGTCCTTCCAATGTCACGAAGAGTAAGAGGATTTGGATGGCTCAGTGTATACC

>Mlu_PSG3

TCTTCCTCTTAACCTTCTGGATCCCGCCCACCACTGCGCGATTTGGTATTGTCTCAACCATTGCTCTTGAAGGGCAGGATGTGATTCTACGTACCCAAAATAGGCCTCCAGGTGTTACAGGCTTTGTTTGGTACAGGGGAAAAGAGATGAACTACTATACTCTTATTGCATCTCTTGCATTGCATTCAAGGAGATCGCTAAGAGGGCCCGAATACAGAGGTCGAGAGACAGTAAACCTTGATGGATCCCTGACCATAAGAAAAGTCACTCTGTGGGACCTAGGAATTTACACCGTAGTAGCCGTCCTTCAAAATTTTCGAAAATAAATTGGAGTTAGACGGCTCATTGTATACT

>Mlu_PSG4

TCTTCCTCTTGTCCTTGTGGATCCCGCCCACCACTGCACAATTTGCTATTCTCTCCACCATTGCTCTTGAAGGGCAGGATGTGATTCTACGTCTCCGCAATCCGCCTCCCGATGTTACAGGGTTTATTTGGTACAGGGGAAGAGAGATGAAGTACCATAATTTCATTGGGTCTCGTGCATGGTATTCCAGTGAATATCTAACAGTTCCCAAATACAGTGGTCGAGAGGAAATAAACCTTGAAGGATCCCTGATCATAAGAAACGTCACTGTGTGGGACTTAGGAATTTACGTCCTAGTAGCCGTCCTTCCAAATTCACGAAGAGAAATAGGATTTGGACAGCTCATTGTATTCC

>Mlu_PSG5

TCTTCCTCTTAACCTTGTGGATCCCGCCCACCACTGCACAATTTGATATTGTCTCGACCATTGCTCTTGAAGGGCAGGATGTGATTCTACGTCTCCGCAATATGCCTCCCGATGTTACAGGGTTTATTTGGTACAAGGGAATGGAGATGAATTACCATAAATTCATTGGGTCTCGTGCATGGTATTCCAGTGAATATCTAACAGGTCCCGAATACAGTGGTCGAGAGGAAATAAACCTTGACGGATCCCTGATCATAAGAAACGTCACACGGAGGGACCAAGATATTTATCTCGTAGTAGCCATCCTTCCAAAGTCACAAAGAGAAATAGGAACTGGAGTGCTCAGAGTATACC

>Mlu_PSG6

TCTTCCTCTTAACCTTGTGGATCCCGCCCACCACTGCACAATTTGATATTGTCTCGACCATTGCTCTTGAAGGGCAGGATGTGATTCTACGTCTCCGTAATACGCCTCCCGATGATACAGGGTTTATTTGGTACAGGGGAATAGAGATGAAGTACCATAAATTCATTGGGTCTCTTGTATCATATTCCAGTGAATATCTAACAGGTCCCAAATACAGTGGTCGAGAGGAAATAACCATTGGAGGACACCTGATCATAAAAAACGTCACTGTGTGGGACCGAGGAAACTACACCGTAATAACCGTCCTTCCAAAGTCACGAAGTGAAATAGGATTTGGAAGGCTCAGAGTATCCCTTGA

>Mlu_PSG7

TCTTCCTCTTAACTCTTTGGATCCCGCCCACCACTGCGCGATTTGCTGTTGTCTCGACCAGTGCTCTTGAAGGGCAGGATGTGATTCTACATACCCACAATAGGCCTCCCAATTGTGCAGGCTTTATTTGGTACAGGGGAGACAAGACGGACTACAATCATTTTATTGCATCTCTTACATTGCATTCAAGGCGATCGGTAAGAGGGCCCAAATACAGTGGTCAAGAGACAGTAAACCTTGATGGATTCCTGACCATAAGAAAAGTCACTCTGAAGGACACAGGAACCTACACCGTAATAGCTGTCCTTGAAAATTCACTAAGAGAAATAGGATGTGGACAGCTCGATGTATACA

>Mlu_PSG8

TCTTCCTCTTAACCTTGTGGATCACGCCCACCACTGCTCGATTTGCTATTGCCTCGACCTTTGCTTTTGAAGGGCATGATGTGATTCTACATCTCCGCAATACACCTCCTGATGTTACAGGGTTTATTTGGTACAGGGGAGAAGAGATGAACTACCATAATTTCATTGGGTCTCGTCCATGGTATTCCAGTGAATATCTAACAGGTCCCAAATACAGTGGTCGAGAGGAAATAAACCTTGATGGAACCCTGATCATAAAAAACGTCACTGCGAGGGACGAAGATACGTATGTCGTAGTAGCCGTCCTTGCAAATTCACAAAGAGTAAGAGGATCTGGATGGCTCAGTATATACC

>Mlu_PSG9

TCCTGCTCTTTACTTTCTGGAGCCGGCCCACTGCTGCCCAACTTGCTATTGTGTCCACCAATGCTGCTGAAGGGGAAGATGTGATTCTACGTATCCGCAATAACCCTCCTGATGCTTCAGGTTATATTTGGTACAGGGGGGAAGGGGCGAAAGCCAGTCGTTATATTGCATTGTTTTCATTGGAGGCAAACGATATTGGAAGAGGGCCTGAATACACTGGTCGAGAGACAATAAACAAAGAAGGATGCTTGCTGATAAAGAAGGTCACCCTGAGAGACACAGGAATCTACACCATAGTAGTCTATCTTCACAATTCAAAAAAGGAAATAGGATTTGGACGGCTCAATGTATACC

>Mlu_PSG10

TTGCACTCTTAAATTTCTGGAGCCTGCCCACCACTGCCCAACTGGCTATTGTGTCAACCAATGCTACCGAAGGACAGGATGTGATTCTACGTATCCTCAATATGCCTCCCCATGTTAGAGGCATCATGTGGTACAGGGGGGAAGAGCCAAACTATGAACATAATATTGCACGTCTTGGAATGTTGCCAGGACATAGAGCAGGGCCTGAACACAGCGGTCGAGAGCAAATAAACTTTGATGGATCCTTGCTGATAAAGAGGGTCACCCTGCAGGACACAGGAAACTACACCATAGTAGTCTACCTTCGAAACTCAAAAAAAGAAATTGGATTTGGACAGCTCAATGTATACG

>Mlu_PSG11

TTTCACTCTTAAATTTCTGGAGCCTGCCCACCACTGCCCAACTTGCTATTGTGTCAACCAATGCTGCCGAAGGGAAGGATGTGACTCTACGTATCCGCAATATGCCTCCTGATTATATAGGCTTCGTGTGGTACAGGGGGGAAGGAGCAAACTACAGACATACCATTGCCAGTCTTTCATTCCGCGTAAGAACTTCTAGAACAGGGCGTGCATACAGCGGTCGAGAGCAAATAAATTTTGATGGATCCTTGCAGATAAAGAGGGTAACCCTGAAGGACACAGGAATCTACACCATAGTAGTCTACCTTCAAGACTATAAGAAAGAAATAGGATTTGGAAGGCTCAATGTATATG

>Mlu_PSG12

TTTCACTCTTAAATTTCTGGAGCCTGCCCACCGCTGCCCAACTCACTTTTGTGTCAACCAAGACTACCGAAGGGAAGAATATGATTCTACTTATCCGCAATATGCCTCTCAATGCTAGAGGCTTCATGGGGTACAGGGGGGAAGGGGCAAACTACAAACATAAAATTGCACGTCTTGGATTGTTCCCAGGACATAGAACAGGGCCTGCATACAGCGGTCAAGAGCAAATAAACTTTGATGGATCCTTGCAGTTACAAAGGCTCACCCAGAATGACACAGGAATCTACATAGTACTACTCTACATTCCCAGCATACTAAAAGAAATAGGATTTGAACGGCTCAATGTATATG

>Mlu_PSG13

TTTCACTCTTAAATTTCTGGAGCCTGCCCACCACTTCCCAACTCTCTATTGTGTCAACCGAAGGACAGGATGTGACTCTACGTATCTGCAATATGCCTCCCAATGCTCAAGGCTACATGTGGTACAGGGGGGAAGGGGCAAACTTCAAACATAAAATTGCAGGTCTTGGATTGTTCCCAGGACATAGAACAGGGCCTGCACACAGCGGTCGAGAGTATATAAACTTTGATGGATTCTTGGTGATAAAGAGGGTCACCCTGGAGGACACAGGAGTCTACACCATAGTAGTATTCCTTCCAGAGCATAAAAAAGAAATAGGATTTGGAGGGCTCAATGTATACG

>Mlu_PSG14

TTTCACTCTTAAATTTCTGGAGCCTGCCCACCACTGCCCAACTCGCTATTGTGTCAACCAATGCTGCCGAAGGACAGGATGTGACTCTACGTATCCGCAATATGCCTCCCAATGCTAAAGGCTTCACGTGGTACAGGGGGGAAGGGGCAAACATCTATCATAATCTTGCAACTCTCGGAACATACCTGAGATTTTATACAACAGGGCCTGCACACAGTGGTCGAGAGCAAATAAACTATGATGGATCCTTGCAATTAAAGCAGGTCACCCAGAAGGACACAGGAATCTACACTGTAGTAGTCTACCTTCCAGGCTGTATAAAAGAAATAGGATTTGGACAGCTCAATGTGTATG

>Mlu_PSG15

TTTCACTCTTAAATTTCTGGAGCCTGCCCACCACTGCCCAACTCGCTATTGTGTCAACCAATGCTGCCGAAGGGAAGGATGTGATTCTAAGTATCCGCAATATGCCTCCCGATGTTAGCAGCTTCATGTGGTACAGGGGGGAAGGGGAAAAATATGAACATAATATTGCACGTCTTGGAATGTTACGAAGACATAGAACAGGGCCTGCATACAGCGGTCGAGAGCGAATAAACTTTGATGGATCCTTGCTGATAAAGAGGGTCACCCAGAAGGACACAGGAATCTACACGGTAGTAGTCTACCTTCCAGACTCAATAAAAGAAATAGGATTTGGACGGCTCAATGTGTACG

>Mlu_PSG16

TTTCACTCTTAAATTTCTGGAGCCTGCCCACCACTGCCCAACTTGCTATTGTGTCAACCAATGCTGCCGAAGGGAAGAATGTGATTCTACATATCCGCAATATGCCTCCTGATCATATAGGCATCGTGTGGTACAGGGGGGAAGGAGCAAACTACAGACATACCATTGCCAGTCATGCATTCCACAGAAGAACTTATAGTATAGGGCGTGCAAACAGCGGTAGAGAGGAAATAAATTTTGATGGATCCTTGCTGATAAAGAGGGTAACCCTGAAGGACACAGGAATCTACACCGTAGTAGTCTACCTTCAAGACTATATAAAAGAAATAGGATTTGGACGGCTCAATGTATATG

>Mlu_PSG17

TTTCACTCTTAAATTTCTGGAGCCTGCCCACCACTGCCCAACTCGCTATTGTTTCAACCAATGCTACCGAAGGGAAGGATGTGATTCTACGTATCCGCAATATGCCTCCCGATGTTAGAGGCTACATGTGGTACAGGGGGGAAGAGGCTAACTACAAACATAATATTGCACGTCTTGGAATATTGCCAGGACATAGAGCAGGGCCTGCACACAGCGGTCGAGAGCAAATAAACTTTGATGGATCCTTGCTGATAAAGAATGTCACCCTGAAGGACACAGGAATCTACACCGTAGTAGTCTACCTTCAAGGCTCAAAAAAAGAAATTGGATTTGGTCGGCTCAATGTATACG

>Mlu_PSG18

TTTCACTCTTAAATTTCTGGAGCCTGCCCACCACTGCCCAACTTGCTATTGTGTCAACCAATGCTGCCGAAGGGAAGGATGTGATTCTACGTATCCACAATATGCCTCCTGATAATATAGGCTTCGTGTGGTACAGGGGGGAAGGAGCAAACTACAGACATACCATTGCCAGTCTTGCATTCCACATAAGAACTTATAGTACAGGGCGTGCATACAGTGGTCGAGAGCAAATAAATTTTGATGGATCCCTGCTGATAAAGAGGGTAACCCTGAAGGACACAGGAATCTACACCGTAGTAGTCTACCTTCGAGACTATAAAAAAGAAATAGGATTTGGACGGCTCAATGTATATG

>Mlu_PSG19

TTTCACTCTTAAATTTCTGGAGCCTGCCCACCACTGCCCAACTCGCTATTGTGTCAACCAATGCTGCGGAAGGACAGGATGTGATTCTACGTATCCGCAATAAGCCTCCCGATGCTACAGGCTTCATGTGGTACAGGGGGGAAGGGGCAATCTACGAACATAATATTGCAAATCTTGGGACTTGGCGAACAGGTAGAACAGGGCCTGCATCCAGTGGTCGAGAGATAATAAACTATGATGGATCCTTGCAGTTAAAGAGGGTCACCCTGAAGGACACAGGAATATACACCGTAGTAGTCTACCTTCCACACTATATAAAAGAAATAGGATTTGGACGGCTCAATGTATATG

>Mlu_PSG20

TCTTCCTCCTAACCTTCTGGATCATGCCCACCACTGCACAATTTGCTATATTCTCGACCGTTGCTTTTGAAGGGCAGGATGTGATTCTACATCTCCGCAATACGCCTCCCAATGTTACACGGTTTATTTGGTACAGGGGAATAGAGATGAAGTACCTTAATTTCATTGGGTCTCGTGAATGGAATTACAGTGAATATCTAAAAGGTCCCGAATACACTGGTCAAGAGGAAATAAACCTTGAAGGATCCCTGATCATAAGAAACGTCACTGTGAGGGACCAAGGTGTTTACATCGTAGTAGCCGTCCTTCCAAAGTCACCAAATGTAAGAAGATTTGGACGGCTCAGAGTATACC

>Mlu_PSG21

TCTTCCTCTTAACCTTCTGGATCCCGCCCACCACTGCGCGATTTGCTATTGTCTCGACCAGTGCTTATGAAGGGCAGGATGTGATTCTATGTCTCTGCAATACGCCTCCTAATGTTACAGGGTTTATTTGGTACAAAGGAATGGACATGAAGTTCCATAATTTCATTGGGTCTCGTCCAAGGAATTACAGTGAATATCTAACAGGTCCTGAATACAGTGGTCGAGAGGAAATACATCTTGAAGGACCCCTGATCATAAGAAACGTCACTGTGAGGGACCAAGGACTTTATATCGTAGTAGCTGTTCTTCCAGAGTCAGGGAGAATAAAAGGATTTGGATGGCTCAGAGTATACG

>Mlu_PSG22

TCCTCCTCTTAACCTTGTGGATCCCGCCCACCACTGCACAATTTGCTATTGTCTCGACCATTGCTCTTGAAGGGCAGGATGTGATTCTACATCTCCGCAATATGCCTACCAATGTTACTGGGTTTATTTGGTACAGGGGAATAGAGATGAAGTACCATAATTTCATTGGGTCTCATGAATGGTATTCCAGTGAATATGTAACAGGTCCTGAATACAGTGGTCGAGAGGAAATAAACCTTGAAGGATCCCTGATCATAAGAAACGTCACTGTGTGGGACCTAGGAATTTACGTCATAGTAGCCGTCCTTTCAAATTCACGAAGAGAAATAGGATTTGGACAGCTCAATGTATTCA

>Mlu_PSG23

TCTTCCTCTTAACCTTCTGGATCATGCCCACCACTGCACAATTTGCTATTGACTCGACCGTTGCTTTTGAAGGGCAGGATGGGATTCTACGTCTCAGCAATAAGCCTCCCAATGTTACAGGGATTATTTGGTACAGGGGAATAGAGATGAAGGTCCTTAATTTCATTGGGTCTCTTGCATGGGATACCAGTGAAATTCTAACAGGTCCCGAATACAGTGGTCGAGAGGAAATAAACCTTGAAGGATCCCTGATCATAAGAAACGTCACTGTACGGGACGAAGGAATTTACGTTGTAGAAGCCGTCCTTCCAAATTCACCAAGAGTAAGAGGATTTGGATGGCTCAGTGTATACC

>Mlu_PSG24

TCTTCCTCTTAACCTTGTGGATCCCGCCCACCACTGCAGAATTTGGTATTGTCTGGAAATTTGCTCTTGAAGGGCAGGATGTGATTCTACGTCTTCGCAATACGCCTCCCGATGTTATAGGGTTTATGTGGTACAGGGGAATGGAGATGACATACCATAATTTCATTGGGTCTCTTGCATGGTATCCCAGGGAATATCTAACAGGTCCCGAATACAGTGGTCGAGAGGAAATAAACCGTGAAGGATCCCTGATCATAAGAAACGTCACTGTGAGGGACCAAGATATTTATGGCGTAGTAGCCGTCCTTCCAAAGTCACGAGGTGTAACAGGACTTGGATGGCTCAGTGTATACA

>Mlu_PSG25

TCTTCCTCTTAACCTTGTGGATCCCGCCCACCACTGCGCGATTTGAGATGGTCATGACCAGTGCTCTTGAAGGGCAGGATGTGATTCTACGTACCCAAAATAGGCCTCCCAATGTTACAGGCTTTATTTGGTACAGGGGATTCGTGATGATCTACAGTAATTTAATTGCATATGTGTCATGGAGTTCAGGGCAATATATAAAAGGGCCCAAATACACTGGTCGAGAGACAATACAATTTAATGGATCCCTGATCATAAGAAACGTCACTATGTGGGACCTAGGATTATACATCGGAGTAGCCGTCCACAACCATTCGAAAACAGAAATAGGATCTGCATTTCTCATTGTATACC

>Mlu_PSG26

TCTTCCTCTTAACCTTGTGGATCCCGCCCACCACTGCACAATTTTCAATTGTCTCGATTAATGCTGCTGAAGGGCAGGATGTGATTCTACATACCCGCAATAGGCCTCCCAGTTGTGCAGGCTTTATTTGGTACAGGGGAGAAAAGAGGGACTATTATCATTTTATTGCATCTGTTGCATGGCGTGTGAGACGATATAGAACAGGGCCCGAATACAGTGGTCGAGAGACAGCAAATCTTGAAGGATCCCTGATCATAAGAAAAGTCACTCTGAAGGACACAGGAACCTACACCGTAATAGCCGTCCTTCAAAATTCACTACGAGAAATAGGATGTGGACAGCTCAATGTATACC

>Mlu_PSG27

TCTTCCTCTTAACCTTGTGGATTCCACCCACCACTGCGCAATTTGAGATTGTCATGACCAGTGCTCTTGAAGGGCAGGATGTGATTCTACGTACCCAAAATAGGCCTCCCAATGTTACAGGCTTTATTTGGTACAGGGGATTCACGAGGAACTACACTAATTTAATTGCATATGTGTCATGGAGTCCAAGGATATATATAACAGGGCCCGCATACAGTGGTCGAGAGATAGTAACATTTGATGGAAACCTAATCATAAGAAACGTCACTGTGTGGGACCTAGGAATCTACATGGCAGTAGCCGTCCTTCACAATTCAAAAACAGTAGAGGGACTTGGATGGCTCAATGTATACC

>Mlu_PSG28

TCTTCCTCTTAACCTTCTGGATCCCGCCCACCACTGCACGATTGGGTATTGTCTTGACCAGTGCTCTTGAAGGGCAGGATGTGATTTTACGTACCCAAAATAGGCCTCAAGGTGTTACAGGCTTTGTTTGGTACAGGGGAATAGAGATGAACTACTATACTCTTATTGCATCTCTTACATTGCATTTAAGGCGATCGTTAAGAGGGCCCGAATACAGTGGTCTAGAGACAGTAAACCTTGATGGATCCCTGACCATAAGAAAAGTCACTGTGTGGGACCTAGGAATGCACATTGTAGTAGCCGTCCTTCAAAATTTACAAAAAGAAATTGGATTTGGACAGCTCAACGTATACC

>Mlu_PSG29

TTTCACTCTTAAATTTCTGGAGCCTGCCCACCGCTGCCCAACTCGCTATTGTGTCAACCAATGCTGCCAAAGGACAGGATGTGACTCTCCGTATCCGCAATATGCCTCCCAATCCTAAAGGCTTCACGTGGTACCGGGGGGAAGGGGCAAACTACTATCATGAACTTGCAAGTCTCGGAACAGTCTTTGGAATTTATATAACAGGTTATGCAAACAGCGGTCGAGAGCAAATAAACTATGATGGATCCTTGCAATTAAAGCAGGTCACCCAGAAGGACACAGGAATCTACACCGTAGTAGTCTACCTTCCAGGCTCAATAAAAGAAATAGGATTTGGACGGCTCAATGTATATG

>Mlu_PSG30

TTTCACTCTTAAATTTCTGGAGCCTGCCCACTGCTGCCCAACTTGGTATTGTGTCAACCAATGCTGCTGAAGGACAGGATGTGACTCTACGTATCCACAATATGCCTCCCAATACTCAAGGCTTAACGTGGTACAGGGGGGAAGGGGCAAACTACTATCATAATCTTGCAACTGTCAGAAAAGACATGAGAAATTTTATAACAGGGTATGCAAACAGCGGTCGAGAGCAAATAAACTATGATGGATCCTTGCAATTAAAGCAGGTCACCCAGAAGGACACAGGAATCTACACCGTAGTAGTCTACCTTCCAGGCAATATAAAAGAAATAGGATTTGGACGGCTCAATGTATATG

>Mlu_PSG31

TTTCACTCTTAAATTTCTGGAGCCTGCCCACCACTGCCCAACTCTCTATTGTGTCAACCAATGCTGCCGAAGGACAGGATGTTACTCTACGTATCTGCAATATGCCTCACAATGCTCAAGGCTACATGTGGTACAGGGGGGAAGGGGCAAACTTCACACATAAAATTGCAGGTCTTGGATTGTTCCCAGGACATAGAACAGGGCCTGCACACAGCGGTCGAGAGTATATAAACTTTGATGGATCCTTGGTGATAAAGAGGGTCACCCTGGAGGACACAGGAATCTACACCGTAGTAGTCTTCCTTCCAGAGCATAAAAAAGAAATAGGATTTGGACCACTCAATGTATACG

>Mlu_PSG32

TTTCACTCTTAAATTTCTGGAGCCTGCCCACCACTGCCGAATTCGTTGTTTTGTCAAAAAATGCTGTCGAAGGGAGGACTGTGATGCTACGTACCCGCGATGTGCCTCCCGGTGTTACTGCCTTCTATTGGTACAAGGGGAGAGAGACATACAACAAAAATATGATTGGACGTCTTACAAAGTTGATAGGAAGACAAAGAACAGGGCCTGCATACAGCGGTCGAGAGTATATATACTTTGATGGAACCTTGGTGATAAATACGGTCACCCAGAAGGACACAGGATTCTACACCTTAGTAGTCAACCTTCCAAACAAAAAACAAGAAATAAGATATGCACGGCTCAATGTATATG

>Mlu_PSG33

TTTCACTCTTAAATTTCTGGAGCCTGCCCACCACTGCCCAACTCGCTGTTGTGTCAACCAATGCTGCTGAGGGACAGGATGTGATTCTACATATCCGCAATATGCCTCCGGAATTTACAGTCTTTGTGTGGTACAGGGGGGAAGGGGAAAACTACAAACATAGTATTGCATGTCTTGGAATATTCCTAAGACATAGAACAGGGCCTGCACACAGCTGTCGAGAGAAAATAAACTTTGATGGATCCTTGCTGATAAAGAGGGTCACCCTGAAGTACACAGGAATCTACACCATAGCAGTCTACCTTCCAGGCTATATAAAAGAAATAGGATTTGGACGGCTCAATGTATACA

>Mlu_PSG34

TTTCACTCTTAAATTTCTGCTGCCTGCCCACCACTGCCCAACTCGCTGTTGTGTCAACCAATGCTGCTGAAGGACAGGATGTGATTCTACATATCCGCAATATGCCTCCGGATGTTAGAGGCTTTGTGTGGTACAGGGGGGAAGGGGAAAACTACAAACATCATATTGCATTTCTTGGAATGTTCCTGTCATATAGAACAGGACCTGCATACAGCGGTCGAGAGCAAGTAAACTTTGACGGATCCTTGCAGATAAAGGGTGTCATCCAGAAGGACACAGGAAACTACACCTTAGTAGCCTACCTTCCAGACTATATAAAAGAAATAGGATTTGGACGGCTCAATGTATACA

>Mlu_PSG35

TCTTCCTCTTAACCATCTGGATCCCGCCCACCACTGCGCGATTTGATATGGTCATGACCAGTGCTGTTGAAGGGCAGGATGTGATTCTATGTACCCAAAATAGGCCTCCCAATGTTACAGGCTTTATTTGGTACAGGGGATTCGTGATGAACTACACTAATTTAATTGCATATGTGTCATGGAGTTCAGGGCAATATATAAAAGGGCCCAAATACACTCGTCGAGAGACAGTAAAATTTAATGGATCCCTGATCATAAGAAACGTCACTATGTGGGACCTAGGATTTTACATCAGAGTAGCCGTCCTCAACAATTCAAAAACAGAAAAAGGATTTGGATTGCTCATTGTATACC

>Mlu_PSG36

TTTCACTCTTAAATTTCTGGAGCCTGCCCACCGCTGCCCAACTCGCTTTTGTGTCAACCAAGACTACCGAAGGGAAGGATGTGATTCTACTTGTCCGCAATATGCCTCTCAATGCTAGAGGCTTCATGGGGTACAGGGGGGAAGGGGCAAACTACAAACATAAAATTGCACGTCTTGGATTGTTCCCAGGACATAGAACAGGGCCTGCATACAGAGGTCAAGAGCAAATAAACTTTGATGGGTCCTTGCAGTTACAAAGGCTCACCCAGAATGACACAGGAATCTACATAGTACTACTCTACATTCCCAGCATACTAAAAGAAATAGGATTTGGACGGCTCAATGTATATG

>Mlu_PSG37

TCTTCCTCTTAACCTCGTGGATCCCGCCCACCACTGCACTATTTGATCCTGAATTTAACTTTGCTCTTGAAGGGAAGGATGTGATTCTACGTCTTCGCAATACGACTCCCGATGTTACAGGGTTTATTTGGTACAGGGGAATGGAGATGGAGTACCCTAATTTCATTGGGTCTCTTGCAAGGGATACCAGTGAATATCTAACAGGTCCCGAATACAGTGGTCGAGAGGAAATAAACCTTGACGGATCCCTGATCATAAGAAACGTCACTGCGAGGGACGAAAATATTTATAGCGTAGTAGCCGTCTTTCCAAATTCACGAAGAGTAAGAGATTTTAGATGGCTCGGTGTATCCC

>Mlu_PSG38

TCTTCCTCTTAACCTTCTGGATCCCGCCCACCACTGCGCGATTTGGTATTGTCTCAACCATTGCTCTTGAAGGGCAGGATGTGATTCTACATACCCAAAATAGGCCTCCAGGTGTTACAGGCTTTGTTTGGTACAGGGGAATAGAGATGAACTACTATACTCTTATTGCATCTCTTACATTGCATTCAAGGCAATCGCTAAGAGGGTCTAAATACAGTGGTCGAGAGACAATAAATCTTGATAGATTTCTGACCATAAGAAAAGTCACTCTGTGGGACATAGGAACGTACACCGTAGTAGCCGTCCTTCAAAATTTTCGAAAAGAATATGGAGTTGGACGGCTCATTGTATACT

>Mlu_PSG39

TCTTCCTCTTAACCTTCTGGATCATGCCCACCACTGCACAATTTGCTATATTCTCGACCGGTGCTTATGAAGGGCAGGATGTGATTCTACATCTCCGCAATAAGCCTCCCCATGTTACAGGGATTATTTGGTACAGGGGAATAGAGATGAAAGTGCATAATTTCATTGGATCTCTTGCATGGGATACCAGTGAATTTCTAACAGGTCCCGGATACAGTGGTCGAGAGGAAATAAACCTTGAAGGATCCCTGATCATAAGAAACGTCACTGTGAGGGACCAAGGAGTTTACGTCGTAGTAGCCATCCTTCCAATGAAACGAAGTGAAACAGGATTGGGATGGCTCAGTGTATACC

>Mlu_PSG40

TCTCACTCTTAACCTTCTGGAGCCCGCCCACCACTGCCCAGCTCGCTATTGTGCCGACCAATGCTGCCGAAGGGAAGGATGTGCTTCTGCGTATCCGCAACAAGCCTCCGGATGCTGTGGGCTTTCTGTGGTACAGGGGGGAAGGGGCGAGATCCCAACGGAATATTGCATCTATTGTAGTGGACTTAAGAGTACACGCATTGGGGCCTGCATACAGCGGTCGAGAGAAAGTAAACAGTGATGGATCCATGCTGTTAAAGAGGGTCACACGGAAGGACACAGGATACTACACCATAGTAGCCCGCCTTCGAGATTCAAAAAAAGAAATAGGATTTGGACAGCTCCGTGTATACC

>Mlu_PSG41

TTTCACTCTTAAATTTCTGGAGCCTGCCCACCGCTGCCCAACTCGCTTTTGTGTCAACCAAGACTACCGAAGGGAAGAATGTGATTCTACTTATCCGCAATATGCCTCTCAATGCTAGAGGCTTCATGGGGTACAGGGGGGAAGGGGCAAACTACAAACATAAAATTGCACGTCTTGGATTGTTCCCAGGACATAGAACAGGGCCTGCATACAGAGGTCAAGAGCAAATAAACTTTGATGGATGCTTGCAGTTACAAAGGCTTACCCAGAATGACACAGGAATCTACATAGTACTACTCTACATTCCCAGCATACTAAAAGAAATAGGATTTGAACGGCTCAATGTATATG

>Mlu_PSG42

TTTCACTCTTAAATTTCTGGAGCCTGCCCACCACTTCCCAACTCTCTATTGTGTCAACCAATGCTGCCGAAGGACAGGATGTGACTCTACGTATCTGCAATATGCCTCCCAATGCTCAAGGCTACATGTGGTACAGGGGGGAAGGGGCAAACTTCACACATAAAATTGCAAGTCTTGGATTGTTCCCAGGACATAGAACAGGGCCTGCACACAGCGGTCGAGAGTATATAAACTTTGATGGATTCTTGGTGATAAAGAGGGTCACCCTGGAGGACACAGGAGTCTACACCATAGTAGTATTCCTTCCAGAGCATAAAAAAGAAATAGGATTTGGACGGCTCAATGTATACG

>Mlu_CC3L1

CCTCACTCTTAACCTTCTGGAGCCTGCCCACCACTGCCCAGCTCACTATTGAATCAGTGCCGCCCAATGCTGCCGAAGGAAAGGATGTGCTTCTCCGTGTCCACAACCTGCCTGGGAATCTTGGAGGCTATGCCTTGGTACAAAGGAGAAATAGTGGACAGCAACCATAAAATTGTATCATATGTAATAGACACTCAAAAAATTACCTACGGACCTGCATACAGTGATCGAGAAAAAATATATCCCAATGGATCCCTGCTGTTTCAGAACGTCACCCGGAAGGACACAGGATACTATATCCTACTAGCCATAGACAAAAATTTTCAGAGCAGACCAGTAACTGGACAGCTCCATGTATACC

>Mlu_CC3L2

CCTTACTCTTAACCTTCTGGAGCCTGCCCACCACTGCCCAGCTCATTATTGAATCAGTGCCGCCCAATGCTGCCGAAGGGAAGGATGTGCTTCTCTGTGTCCACAACCTGCCTGGGGATCTATTCGGCTATACCTGGTACAAAGGGGAAATAGTGGACTACAGCCGTCGAATCATATCATATGTAGTAGACACTCAAACAGCTACCCTCGGGCCTGCAAGCAGTGGTAGAGAGACAATATACCCCAATGGATCCCTGCTGTTCCAGAACGTCACCCTGAAGGACACAGGATACTACACCCTACAAGCCACAGTGAAAGATTTACAGAGCAAACAAGTAACTGGACAGCTCCGTGTATACC

>Mlu_CC3L3

CCTCACTCTTAACCCTCTGGAGCCTGCCCACCACTGCCCAGCTCACTATTGAATCAGTGCCGCCCAATGCTGTTGAAGGGAAGGATGTGCTTCTCTGTGTCCACAACCTGCCTGGGAATCTTGGAAGCTATACCTGGTACACAGGGGGAAAGGTGGACTACAGCCATCAAATTGTATCATATGTAATAGACACTCAAACAACTACCCGTGGGCCTGAAAGCAGTGGTAGAGAGACAATATACCCCAATGGATCCCTGCTGTTCCAGAAAGTCTCCCTGAAAGACACAGGATACTATACCCTACTAGCCACAGACAAAGATTATGAGAGCATGCAAGTAACTGGACAGCTTCGTGTATACC

>Mlu_CC1L1

CCTCACTCTTAACCTTCTGGAGCCTGCCCACCACTGCCCAGCTCACTATTGAATCAGTGCCGCCCAATGCTGCCGAAGGGAAGGATGTGCTTCTCCGCGTCCACAATCTGCCTCGGAATCTAATGGGCTATATGTGGTACAAAGGAGAAATAGTGGGCGGTGACCTTCAAATTGTATCATATGTAATAGACACTCAAACAACTAGTCTCGGGCCCGCATACAGCCATCGAGAGACAATATACCCCAATGGATCCCTGCTGTTCCAGAAAGTCACCCTGCAGGACACCGGATACTACACCCTACAAGCCGTAGACAAAGATTTTCGGAGCAAAAGAGTAACTGGACAGATCCGTGTTTACC

>Mlu_CC1L2

CCTCACTCTTAACCTTCTGGAGCCTGCCCACCACTGCCCAGCTCACTATTGAATCAGTGCCGCCCAATGCTGCCGAAGGGAAGGATGTGCTTCTCCGTGTCCACAACCTGCCTGGGGATCTTGGAGGCTTTATCTGGTACAAAGGGGAAATAGTGGACAGTGACCTTCAAATTGTATCATATGTAATAGACACTCAAACAATTACCCTCGGGCCCGCATACAGCCATCGAGAGACAATATACCCCAATGGATCTTTGCTGTTCCAGAAAGTCACCCTGCAGGACACAGGATACTACACCCTACTAGCCATGAAGAAAATTTATACCAGCATACAAGTAACTGGACAGCTCCGTGTATACC

>Mlu_CC1L3

CCTCACTCTTAACCTTCTGGAGCCTGCCCACCACTGCCCAGCTCACTATTGAATCAGTGCCGCCCAATGCTGCCGAAGGGAAGGATGTGCTTCTCCGTGTCCACAACCTGCCTGGGAATCTATTCGGCTATACCTGGTACAAAGGGGAAATAGTGTACAGCAGCCGTCTAATCGTATCATATATAATAGACACTCAAACAACTACCTTTGGGCCTGCAAACAGTGGTAGAGAGACAATATACCCCAATGGATCCCTGCTGTTCCAGAACGTCACCCTGAACGACACAGGATACTACACCCTTAAAACCACAGGGAAAGATTTACAGGAGAAAAAAGTAACTGGACAGCTCCGTGTATACC
